# Selective Tuning of Benzothiadiazole Functionality Enables High Crystallinity and Mobility in Regiorandom *n*-Type Polymers for Organic Field-Effect Transistors

**DOI:** 10.1021/acs.macromol.4c02854

**Published:** 2025-03-21

**Authors:** Panagiota Kafourou, Qiao He, Xiantao Hu, Mohamad Insan Nugraha, Wen Liang Tan, Joel Luke, Bowen Ding, Christopher R. McNeill, Thomas D. Anthopoulos, Martin Heeney

**Affiliations:** † KAUST Solar Center, Physical Sciences and Engineering Division (PSE), King Abdullah University of Science and Technology (KAUST), Thuwal 23955-6900, Kingdom of Saudi Arabia; ‡ Department of Chemistry and Centre for Processable Electronics, Imperial College London, London W12 0BZ, U.K.; § Department of Materials Engineering, Monash University, Clayton, Victoria 3800, Australia; ∥ Research Center for Nanotechnology Systems, National Research and Innovation Agency (BRIN), South Tangerang, Banten 15314, Indonesia; ⊥ Collaboration Research Center for Advanced Energy Materials, National Research and Innovation Agency − Institut Teknologi Bandung, Jl Ganesha 10, Bandung 40132, Indonesia; # Henry Royce Institute and Photon Science Institute, Department of Electrical and Electronic Engineering, The University of Manchester, Oxford Road, Manchester M13 9PL, U.K.

## Abstract

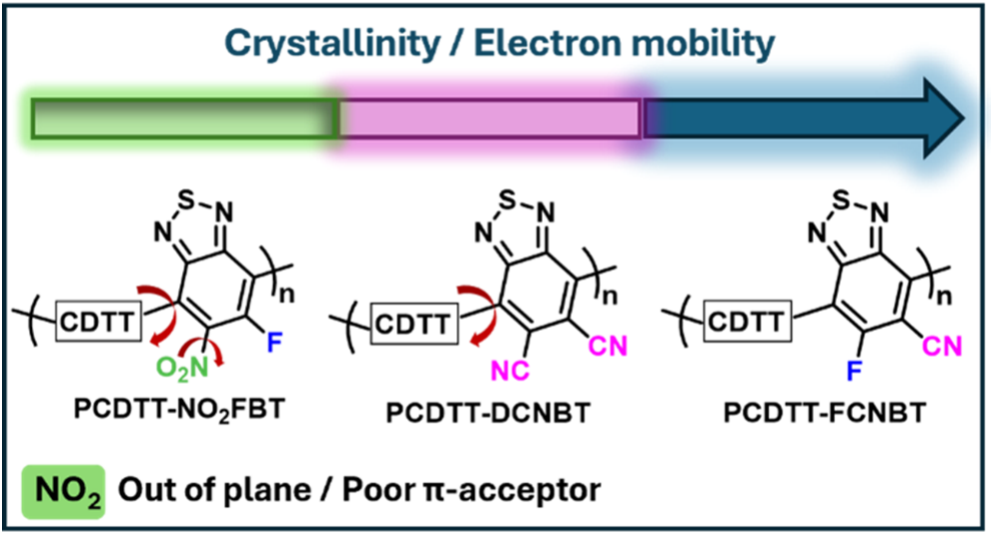

We report three novel donor–acceptor (D–A) copolymers sharing a common fused donor unit (CDTT) but differing in the functionalization of the benzothiadiazole (BT) acceptor unit. Acceptors bearing two cyano groups (DCNBT) are compared to novel acceptors bearing one cyano and one fluorine group (FCNBT) or one nitro and one fluoro group (NO_2_FBT). The choice of the acceptor has a significant effect on the optoelectronic properties of the resulting polymers. In organic field-effect transistor (OFET) devices, PCDTT-DCNBT exhibited moderate performance with an electron mobility of 0.031 cm^2^ V^–1^ s^–1^, whereas PCDTT-FCNBT demonstrated significantly improved electron mobility (0.4 cm^2^ V^–1^ s^–1^). The improved performance is attributed to increased backbone linearity combined with a more coplanar backbone and high thin-film crystallinity. In comparison, the presence of the nitro group is shown to have a detrimental impact, with a blue-shifted absorption and a 0.2 eV increase in band gap compared to the cyanated polymers. Steric effects are shown to limit the nitro group’s π-accepting capability and result in reduced device performance, with an electron mobility of 0.024 cm^2^ V^–1^ s^–1^. This study introduces a new BT building block and highlights that substituent tuning via cyano and fluorine groups is an effective approach for modulating polymer morphology and electron transport.

## Introduction

Semiconducting polymers are attractive components for cost-effective solution-processed organic solar cells (OSCs), biosensors, and organic field-effect transistors (OFETs).^1−4^ For OFET circuits, both *p*-type (hole transporting) and *n*-type (electron transporting) semiconductors are required to ensure high noise immunity and low static power consumption.^5^ At present, the performance of *n*-type polymers lags behind *p*-type semiconductors due to various factors, such as increased synthetic complexity, limited electron-deficient building blocks, and more problematic air stability.^5,6^ A common system used for the development of *n*-type polymer semiconductors is the donor–acceptor (D–A) structure that includes alternating electron-rich, donor (D), and electron poor, acceptor (A), units.^7^ The hybridization of molecular orbitals of such systems leads to narrowing of the band gap, and the selection of donor and/or acceptor units is crucial for tuning the higher occupied and lower unoccupied molecular orbitals (HOMO/LUMO) of the resulting semiconductor. Low-lying LUMO levels are important to facilitate electron injection and improve operational stability in the presence of water and oxygen.^8−10^ Acceptor (A) comonomers are often electron-poor aromatics containing electron-withdrawing functional groups, like fluorine, or π-acceptors, such as cyano groups, and are crucial for determining the *n*-type properties of the conjugated polymer.^5,11^ More specifically, control of *n*-type behavior is significantly affected by the π* energy of the individual A unit, which strongly influences the LUMO of the polymeric system.

**Scheme 1:**
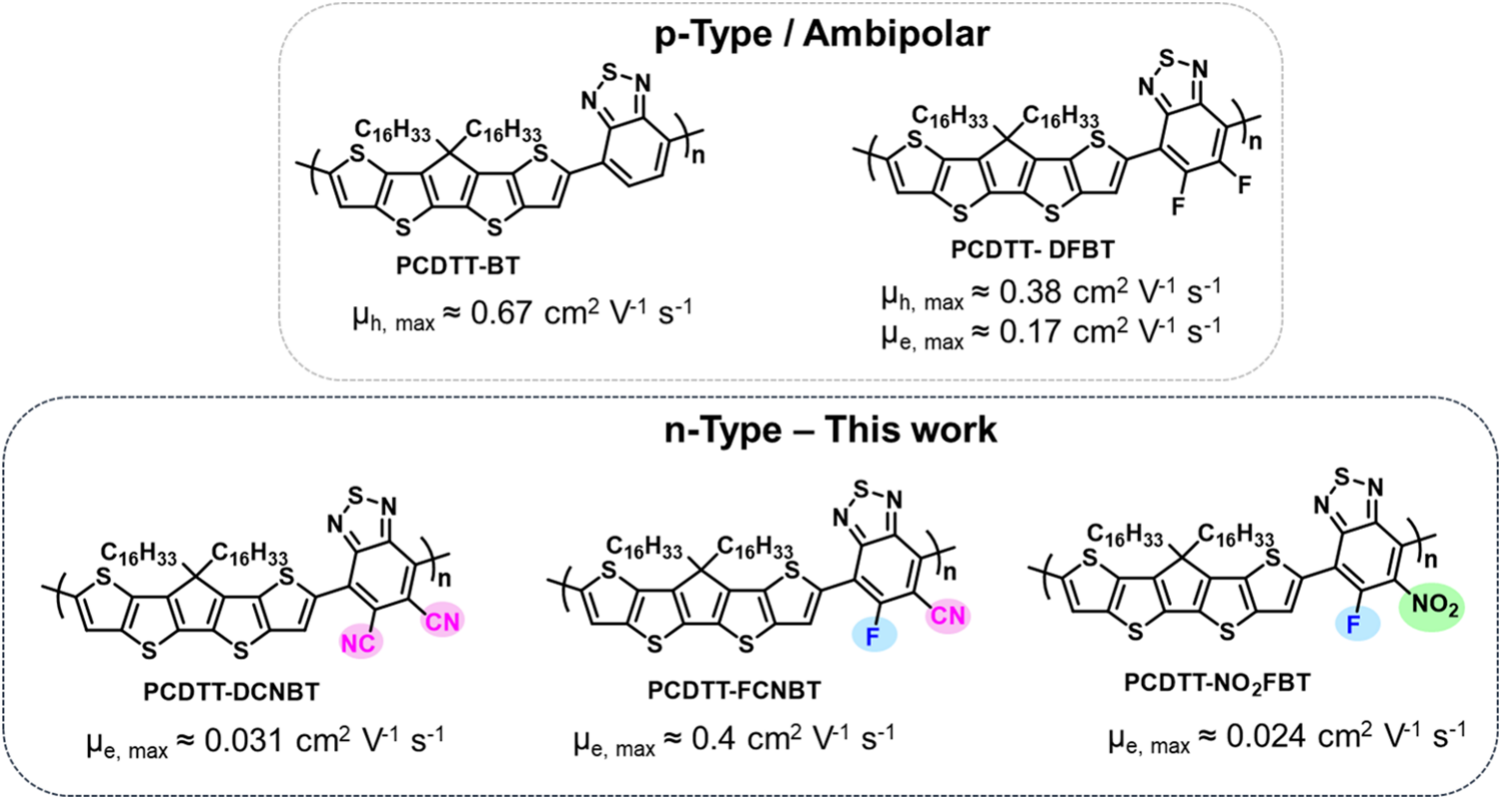
(Top) PCDTT-BT Polymers as Reported in the Literature and (Bottom) Our Work on Functionalization of the BT Monomer to Attribute *n*-Type Properties on CDTT-Based Polymers

Bridged bithiophenes have been commonly employed as electron-rich donor units in conjugated polymers.^12,13^ The bridging in these units enforces thiophene coplanarity, which enhances electron delocalization and reduces reorganization energy of the polymer. Cyclopenta [2,1-*b*;3,4-*b*]dithiophene (CDT),^14,15^ in which bithiophene is bridged by one carbon, has been extensively used in both OSCs^16,17^ and OFETs.^16,18,19^ An extended analogue of CDT, 9H-thieno[3,2-*b*]thieno[2″,3″:4′,5′]thieno[2′,3′:3,4]cyclopenta-[1,2-*d*]thiophene (CDTT), in which bis(thieno[3,2-*b*]thiophene) is fused by a bridging carbon atom, has been developed to further enhance conjugation length and promote intermolecular packing.^20,21^ Copolymers of CDTT with 2,1,3-benzothiadiazole (BT) have demonstrated promising performance in OFET devices, exhibiting unipolar *p*-type behavior, with a hole mobility up to 0.67 cm^2^ V^–1^ s^–1^. Increasing the electron affinity of the BT comonomer by fluorination (5,6-difluoro-2,1,3-benzothiadiazole, DFBT) resulted in a polymer, PCDTT-DFBT, with a lower lying LUMO level that exhibited ambipolar performance in OFET devices, with a hole mobility of 0.38 cm^2^ V^–1^ s^–1^ and electron mobility of 0.17 cm^2^ V^–1^ s^–1^ (Scheme 1, top).^20^

In our search for *n*-type polymers based on CDTT, we hypothesized that the use of a stronger electron-accepting comonomer than DFBT could enhance electron transport by lowering the LUMO level, thereby promoting electron injection from common electrode materials. Replacement of the fluorine groups of DFBT with two cyano groups is known to give the much stronger acceptor 5,6-dicyano-2,1,3-benzothiadiazole (DCNBT).^22−29^ While this has previously been shown to promote *n*-type transport, it can also result in an undesirable disruption to backbone planarity due to the larger size of the cyano group compared to fluorine, which results in steric interactions with adjacent comonomers.^23^ To compensate for this, we designed a new, unreported BT monomer containing one cyano group and one fluorine group. The resulting monomer, 6-fluoro-5-cyano-2,1,3-benzothiadiazole (FCNBT), benefits from the combination of one fluorine group, facilitating backbone planarity,^30,31^ and one cyano group, acting as a strong π-acceptor. As a comparison to the cyano in FCNBT, we also synthesized the monomer 5-fluoro-6-nitrobenzo-2,1,3-thiadiazole (NO_2_FBT) that utilizes a nitro group, to understand the impact of different π-acceptors on the structure and *n*-type properties of the polymer. Thus far, despite their strong acceptor character, nitro groups have rarely been investigated as π-acceptors in D–A polymer semiconductors. The use of nitrated BT derivatives as comonomers is hard to find,^32−34^ despite the widespread prevalence of nitrated BT derivatives as precursors to quinoxaline and various fused carbazoles as materials for low band gap polymers. However, the utility of the nitro group has been explored in other systems. For example nitrated-pentacene derivatives have been used as acceptor materials in OSCs yielding lower lying LUMO energy levels when compared to other π-acceptors, including cyano groups.^35^ A copolymer of 3,4-dinitrothiophenes and diketopyrrolopyrrole (DPP) has been reported as an *n*-type polymer for transparent OFETs.^36^ Nitrated fluorenone derivatives have also been reported, achieving modest electron mobilities (10^–4^ cm^2^ V^–1^ s^–1^) in OFETs.^37,38^

We hereby report three novel *n*-type D–A polymers, PCDTT-DCNBT, PCDTT-FCNBT and PCDTT-NO_2_FBT (Scheme 1), in which the CDTT core is copolymerized with DCNBT, FCNBT, and NO_2_FBT. By comparing the optoelectronic properties and structural characteristics of the three polymers, we demonstrate that PCDTT-FCNBT shows a similar optical response in the solid state being highly ordered and crystalline when compared to PCDTT-DCNBT. The polymer PCDTT-NO_2_FBT showed a less ordered structure, with the nitro group strengthening the electron-withdrawing effect but not facilitating planarization through intramolecular interactions along the backbone. All three polymers demonstrated electron transport behavior in transistor devices with PCDTT-FCNBT exhibiting the highest electron mobility at μ_e,max_ ≈ 0.4 cm^2^ V^–1^ s^–1^ followed by PCDTT-DCNBT with moderate electron mobility at μ_e,max_ ≈ 0.031 cm^2^ V^–1^ s^–1^ and PCDTT-NO_2_FBT at μ_e,max_ ≈ 0.024 cm^2^ V^–1^ s^–1^.

### Synthesis

**Scheme 2:**
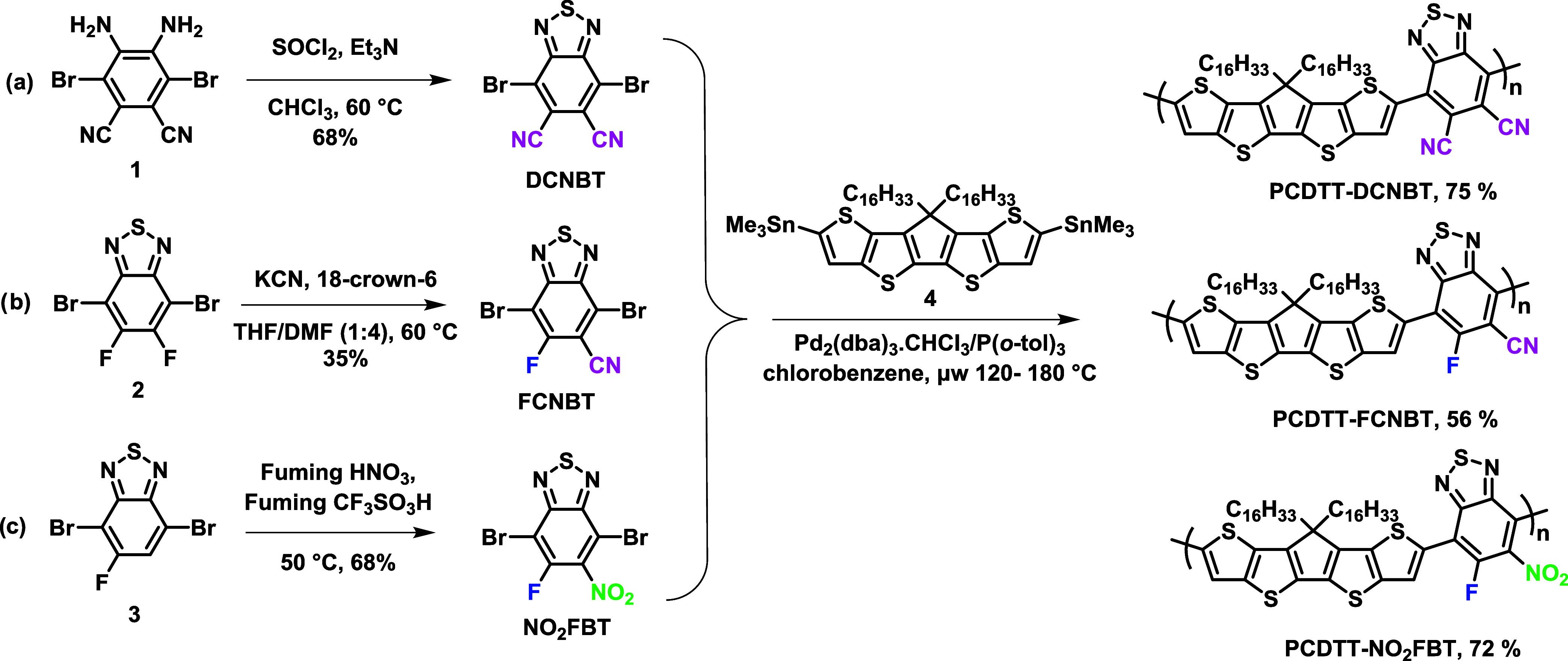
Synthesis of Monomers DCNBT, FCNBT, and NO_2_FBT and Polymers PCDTT-DCNBT, PCDTT-FCNBT, and PCDTT-NO_2_FBT

The DCNBT monomer was synthesized following a modification of the reported protocol (Scheme 2).^39^ Thus, intermediate 4,5-diamino-3,6-dibromopthalonitrile (1) was prepared by reaction of commercially available 4,5-diaminopthalonitrile with potassium bromide and hydrobromic acid in the presence of *tert*-butyl hydroperoxide in good yields over 78%.^40^ Subsequent reaction with thionyl chloride in the presence of triethylamine afforded DCNBT in 68% yield after column chromatography.^41^ FCNBT has not previously been reported but was readily prepared via controlled aromatic nucleophilic substitution, albeit in low isolated yield (10%), from the reaction of commercially available 4,7-dibromo-5,6-difluorobenzo[*c*][1,2,5]thiadiazole (2) with KCN and 18-crown-6 in a mixture of THF and DMF, with starting material recovery at 76% yield. Accounting for the unreacted starting material, which was separated by chromatography and reused, the yield of the product was 35%. Several other conditions were investigated to try to improve the yield, including the use of alternative aprotic solvents such as DMSO, DMAc, and NMP and varying the temperature from 40–60 °C but no improvements in yield were observed. Monomer NO_2_FBT was prepared by nitration of commercially available 4,7-dibromo-5-fluorobenzo[*c*][1,2,5]thiadiazole (3) with a mixture of concentrated nitric acid and concentrated triflic acid. The reaction proceeded smoothly, with the product isolated in a 61% yield.

Polymers were prepared by the reaction of distannylated CDTT (4)^19^ with all three monomers via Stille polymerization under microwave heating. After 50 min of microwave heating with a gradual increase in the temperature from 120 to 180 °C, the polymers were precipitated into methanol and the resulting precipitate purified by sequential Soxhlet extraction to remove the catalyst and low-molecular-weight (MW) oligomers. Soxhlet was performed as follows: methanol, acetone, hexane, chloroform for the polymer PCDTT-NO_2_FBT and for polymers PCDTT-FCNBT and PCDTT-DCNBT, further extraction with chlorobenzene was required to extract most of the material. For PCDTT-DCNBT, further extraction of the remaining material with *o*-dichlorobenzene afforded a (presumably) higher weight polymer, but this was very poorly soluble after concentration and precipitation, precluding further analysis. The molecular weights of the resulting polymers were determined by gel permeation chromatography (GPC) against polystyrene standards, as shown in Table 1.

**Table 1:** Summary of Molecular Weights and Optical and Electronic Properties of Polymers

	*M_n_* (kDa)^a^	(Đ)	*E*_(DFT)_ (HOMO, eV)^b^	*E*_(DFT)_ (LUMO, eV)^b^	*E*_(PESA)_ (HOMO, eV)^c^	*E*_ox,onset_ (V) (HOMO, eV)	*E*_red,onset_ (V) (LUMO, eV)^d^	λ_max_, nm (sol)	λ_max_, nm (film)	*E*_g_, (eV) (sol, opt)^e^	*E*_g_, (eV) (film, opt)^f^
PCDTT-DCNBT	20.1	1.4	–5.0	–3.5	–5.48	0.42 (−5.22)	–0.82 (−3.98)	820	862	1.33	1.31
PCDTT-FCNBT	56.9	1.6	–4.8	–3.3	–5.40	0.54 (−5.34)	–1.23 (−3.57)	780	861	1.43	1.32
PCDTT-NO_2_FBT	33.9	2.0	–4.8	–3.2	–5.51	0.60 (−5.40)	–1.30 (−3.50)	721	755	1.51	1.45

a PCDTT-DCNBT, PCDTT-FCNBT, and PCDTT-NO_2_FBT.

b Simulations were carried out on single molecules in the gas phase at the B3LYP level of theory with the basis set 6-31G(d,p).

c PESA estimated HOMO levels for as-cast films. Error estimated as ±0.05 eV.

d HOMO/LUMO levels were estimated from the onset of first oxidation/reduction and referenced against ferrocene/ferrocenium at −4.8 eV. Error ±0.1 eV.

e Estimated from the absorption onset in solution with error ±0.01 eV.

f Estimated from the absorption onset with error ±0.01 eV.

### Optoelectronic Properties

**Figure 1:**
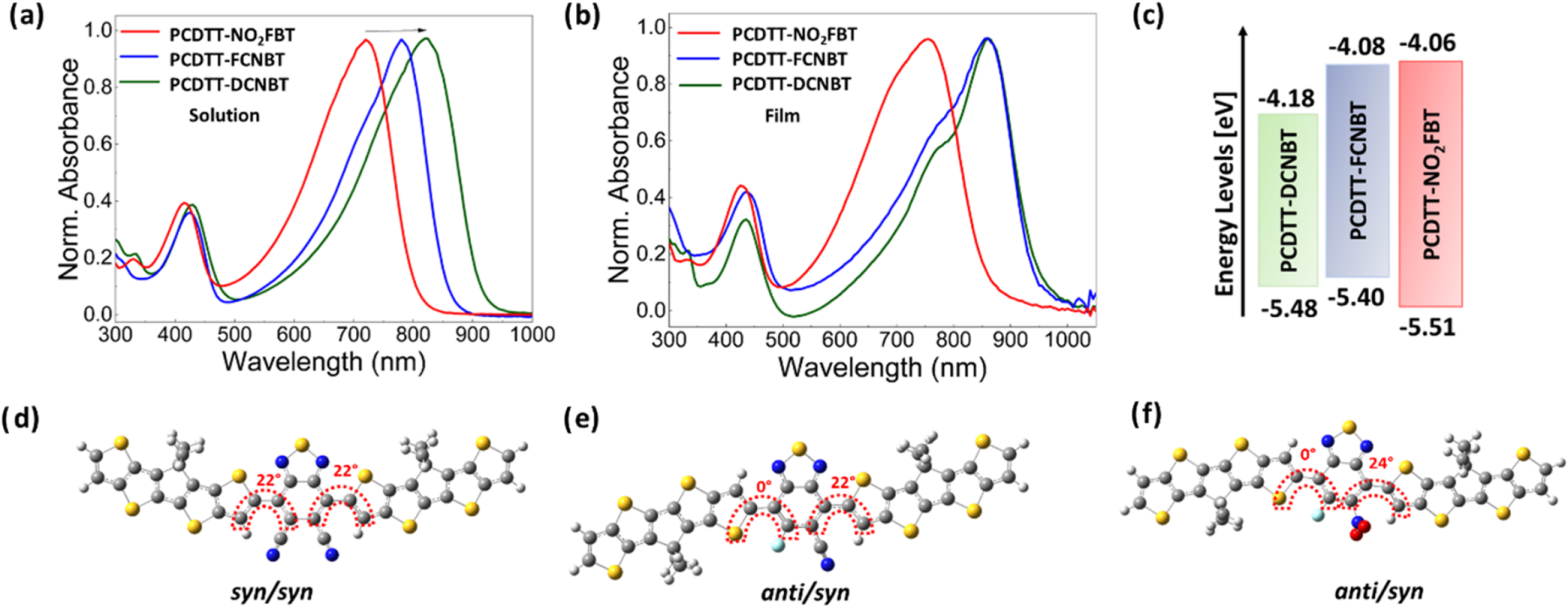
Comparison of optical properties of polymers. UV–vis spectra in (a) chlorobenzene solution and (b) as-spun-cast thin films. Films were cast from *o*-dichlorobenzene (PCDTT-DCNB), chlorobenzene (PCDTT-FCNBT), and chloroform (PCDTT-NO_2_FBT). (c) Schematic diagram of the electronic energy levels derived from PESA and the optical band gap. (d–f) DFT-derived optimized geometries with dihedral angle of lowest energy conformation. *Syn* and *anti* refer to the respective orientations of sulfur atoms in the CDTT and BT. Simulations were carried out on single molecules in the gas phase at the B3LYP level of theory with the basis set 6-31G(d,p).

The UV–vis absorption spectra of the polymers in chlorobenzene solution and as-spun thin films are shown in Figure 1. Note that the films were spun from different solvents in each case since these were visually found to afford the best-quality films. However, changing solvent did not significantly change the film spectra, as shown in Figure S2. Polymer PCDTT-DCNBT (Figure 1a,b green) has an absorption maximum at 820 nm in solution that is red-shifted by 42 nm in the solid state, including a clear vibronic shoulder at around 779 nm. The band gaps extracted from the absorption onset are 1.33 and 1.31 eV for solution and solid state, respectively. Reducing the strength of the electron-accepting unit by replacing a cyano group with a fluorine results in a blue shift in absorption in solution, with PCDTT-FCNBT (Figure 1a,b blue) absorbing at 780 nm. The band gap was calculated at 1.43 and 1.32 eV in solution and solid states, respectively. Interestingly, in the solid state, PCDTT-FCNBT and PCDTT-DCNBT have identical absorption maxima owing to the larger shift (80 nm) upon film formation for PCDTT-FCNBT. Polymer PCDTT-NO_2_FBT (Figure 1a,b red) shows a solution absorption maximum at 721 nm that is red-shifted by 34 nm in the solid state, with less defined vibronic features. The band gap was calculated at 1.51 and 1.45 eV in solution and solid state, respectively.

The larger bathochromic shift of PCDTT-FCNBT compared to PCDTT-DCNBT upon solidification suggests that the inclusion of one fluorine atom leads to more effective backbone planarization, as well as enhanced intra- and intermolecular interactions that have a significant effect on the optical response of the material. Unexpectedly, the inclusion of a nitro group (PCDTT-NO_2_FBT) does not reduce the band gap of the material in comparison to that of the cyano analogue (PCDTT-FCNBT). This is somewhat surprising since nitro groups are generally thought to be more electron-withdrawing than cyano groups. For example, by comparison of their Hammett parameters, it can be seen that the nitro group is more deactivating than cyano; σ_para_ 0.78 for NO_2_ and 0.66 for CN; σ_meta_ 0.71 for NO_2_ and 0.56 for CN.^42^ However, as we discuss below, we attribute this to steric effects that twist the nitro group out of planarity, and therefore out of conjugation, with the BT, thereby reducing electron-withdrawing resonance effects (Figure 1f).

The frontier molecular orbital energy levels of all three polymers were estimated by cyclic voltammetry (CV), which was referenced against ferrocene (Figure S2). CV allowed for a direct experimental measurement of the reduction potential, which can be correlated to the LUMO energy. However, CV can be complicated by the presence of the solvent and electrolyte, which can potentially penetrate the film during measurements, changing the energetics. Therefore, photoelectron spectroscopy in air (PESA) was also used to measure the ionization potential of thin films. In order to compare between techniques, the ionization potential (PESA) and oxidation/reduction onsets (CV) were converted to HOMO/LUMO levels. Polymer films for PESA were prepared by spin coating, and films for CV were prepared via drop casting on a platinum rod working electrode and measured in a degassed solution of tetra-*n*-butylammonium hexafluorophosphate (0.1 M) in acetonitrile.

HOMO energy levels using PESA were estimated at −5.48 −5.40, and −5.51 eV for PCDTT-DCNBT, PCDTT-FCNBT, and PCDTT-NO_2_FBT, respectively. The measurements suggested changing a cyano group to a less electron-withdrawing fluorine group resulted in an increase in the HOMO energy, whereas changing to a nitro group resulted in a little change. Measurements on thermally annealed films (Figure S2b) were identical, within experimental error, suggesting that despite the changes observed by X-ray crystallography, the energetics do not change upon heating.

CV showed shallower HOMO energy levels compared to those of PESA, likely due to the different experimental conditions. All three polymers demonstrated excellent electrochemical stability during three cycles, as shown in Figure S2a. Of particular note is the stability of the reduction cycle, given the desired application as *n*-type materials. Both PCDTT-DCNBT and PCDTT-NO_2_FBT exhibited two quasi-reversible reduction peaks, whereas PCDTT-FCNBT only exhibited a single reversible peak. The energy levels were estimated from the onset of first oxidation (*E*_ox,onset_) and first reduction (*E*_red,onset_) peaks and are summarized in Table 1. The trend in LUMO levels is relatively clear, with PCDTT-DCNBT exhibiting the deepest LUMO. Changing a cyano to a fluorine or nitro group increased the LUMO. The HOMO levels demonstrated a different trend to the PESA measurements, with PCDTT-DCNBT exhibiting the shallowest, followed by PCDTT-FCNBT and then PCDTT-NO_2_FBT. It is notable that the oxidation onset is less sharp for PCDTT-DCNBT (Figure S2a) than for the other two polymers, and it is possible that the lower molecular weight of PCDTT-DCNBT has an influence here. While the weight appears sufficient to be above the effective conjugation length, the lower relative value may result from a different morphology and changed swelling of the film, which may influence penetration of the electrolyte/solvent into the film, thereby influencing energetics. Therefore, the overall energetics (Figure 1c) were estimated using the thin-film I.P. measured by PESA, in combination with the optical band gap.

To compare the molecular conformations of the three polymers, the minimum energy structures were calculated with density functional theory (DFT) using the B3LYP functional and a 6-31G(d,p) basis set. To ensure a global minimum energy conformation was found, relaxed potential energy scans (PES) were also performed, in which the angles between the BT and CDTT units were scanned between 0 and 180° (Figure S3). When a cyano or nitro group was present, the minimum energy structures of the polymers adopt a nonplanar configuration, due to interactions between these π-acceptor groups on the BT and the adjacent thienyl end groups of the CDTT units. Twist angles of 22 and 24° between the CDTT and the BT were found for the cyano- and nitro groups, respectively, with the lowest energy conformer being that where the sulfurs of BT and CDTT were *syn* in both cases. However, we note that the energetic penalty for planarization is relatively small (<3 KJ mol^–1^). Conformers in the *anti* conformer exhibited a higher penalty to planarization, around 5–7 KJ mol^–1^, and the deviation from coplanarity is even more pronounced for the nitro group in the less preferred *anti* conformer.

On the side of fluorine, the BT and CDTT units adopt a near-planar arrangement, with an *anti*-conformation preferred but with a coplanar arrangement also observed in the slightly higher energy *syn* conformer. This preferred conformation results in a more linear backbone for both PCDTT-FCNBT and PCDTT-NO_2_FBT compared to PCDTT-DCNBT, which exhibits a notable curvature (Figures 1d–f and S4). The backbone curvature has previously been to result in reduced overlap of conjugated backbones and nonoptimal charge transfer characteristics.^43−45^

In addition, a PES scan of the NO_2_ group was also performed to look at its contribution to the π-system. As seen in Figure S5, the minimum energy is found when the NO_2_ group is twisted away from planarity with the BT unit, to minimize interactions with the adjacent CDTT. This results in conjugation between the π system of the NO_2_ group and the BT being disrupted, and therefore the electron-withdrawing effect of the nitro becomes an inductive effect, rather than a combination of resonance and induction. This explains the reduced effect of the nitro group in narrowing the band gap compared with the cyano group, which combines both the resonance and inductive effect.

The simulated HOMO/LUMO energy levels for trimer species (Table 1) show reasonable agreement with the trend of the experimental data in the case of LUMO, agreeing that PCDTT-DCNBT is the easiest to reduce. The trend of the HOMO is less clear-cut, predicting that PCDTT-DCNBT should be deeper lying than the other two polymers.

### OFET Performance

**Figure 2:**
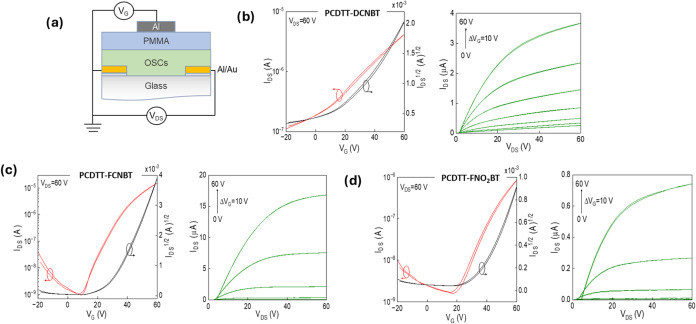
Transfer and output curves for (a) PCDTT-DCNBT annealed at 200 °C, (b) PCDTT-FCNBT annealed at 250 °C, and (c) PCDTT-NO_2_FBT annealed at 120 °C.

The semiconducting properties of the three polymers were investigated in top-gate bottom-contact transistors, which employed Au source and drain electrodes, Al gate electrodes, and a poly(methyl methacrylate) (PMMA) gate dielectric (Figure 2). A layer of aluminum (5 nm) was deposited before the Au (45 nm) to improve electrode adhesion. As discussed above, due to variations in the polymers’ solubility, different solvents were used to afford the visually smoothest films with *o-*dichlorobenzene (DCB) for PDCNBT-CDTT, chlorobenzene (CB) for PFCNBT-CDTT, and chloroform (CF) for PNO_2_FBT-CDTT films. Although solvent variation can influence the device performance for some polymers, here, we note that varying the solvent did not impact the thin-film UV–vis spectra (Figure S1) or the ionization potential as measured by PESA (Figure S6). Given the films all required annealing to afford optimal performance, we believe any variation in device performance arising from the solvent has a minor impact.

Annealing the semiconducting polymer film had a large impact on device performance, as summarized in Figure S7. Individual transfer and output plots for all devices as a function of temperature are shown in Figure S9–11. All polymers were annealed before deposition of the gate dielectric and gate electrode, and in all cases, the PMMA dielectric was dried at 90 °C to avoid leakage issues. An arbitrary annealing temperature of 120 °C was initially chosen, and measurements were then increased in 30 °C intervals, up to 250 °C. The mobility of PCDTT-DCNBT initially increases with temperature, peaking with annealing at 200 °C, before decreasing at higher temperatures. The maximum electron mobility (μ_e,max_) was 0.031 cm^2^ V^–1^ s^–1^, with an on/off ratio of 5 × 10^2^, and a low threshold voltage of *V*_T_ ≈ 1.5 V. The performance of PCDTT-FCNBT shows a similar trend, increasing with annealing temperature, reaching μ_e_ ≈ 0.22 cm^2^ V^–1^ s^–1^ at 180 °C, followed by a decrease if annealed at 200 or 220 °C. Surprisingly, annealing to 250 °C resulted in a further increase in performance, reaching a μ_e,max_ ≈ 0.4 cm^2^ V^–1^ s^–1^. This indicates a clear impact of the morphology on defining the charge transport properties of PCDTT-FCNBT. At this peak mobility, both the on/off ratio (10^4^) and threshold voltage (*V*_T_ ≈ 25 V) are larger than those of PCDTT-DCNBT. Lastly, polymer PCDTT-NO_2_FBTshowed an optimum annealing temperature at 120 °C with electron mobility dropping significantly at higher annealing temperatures. Polymer PCDTT-NO_2_FBT demonstrated the lowest electron mobility, μ_e,max_ ≈ 0.015 cm^2^ V^–1^ s^–1^, with an on/off ratio of 10^4^, and *V*_T_ ≈ 30 V. When annealed at the highest temperature of 250 °C, the device performance degraded completely, possibly due to some degradation. Table S1 summarizes the mobility in relation to annealing temperature.

### Structural Analysis

**Figure 3:**
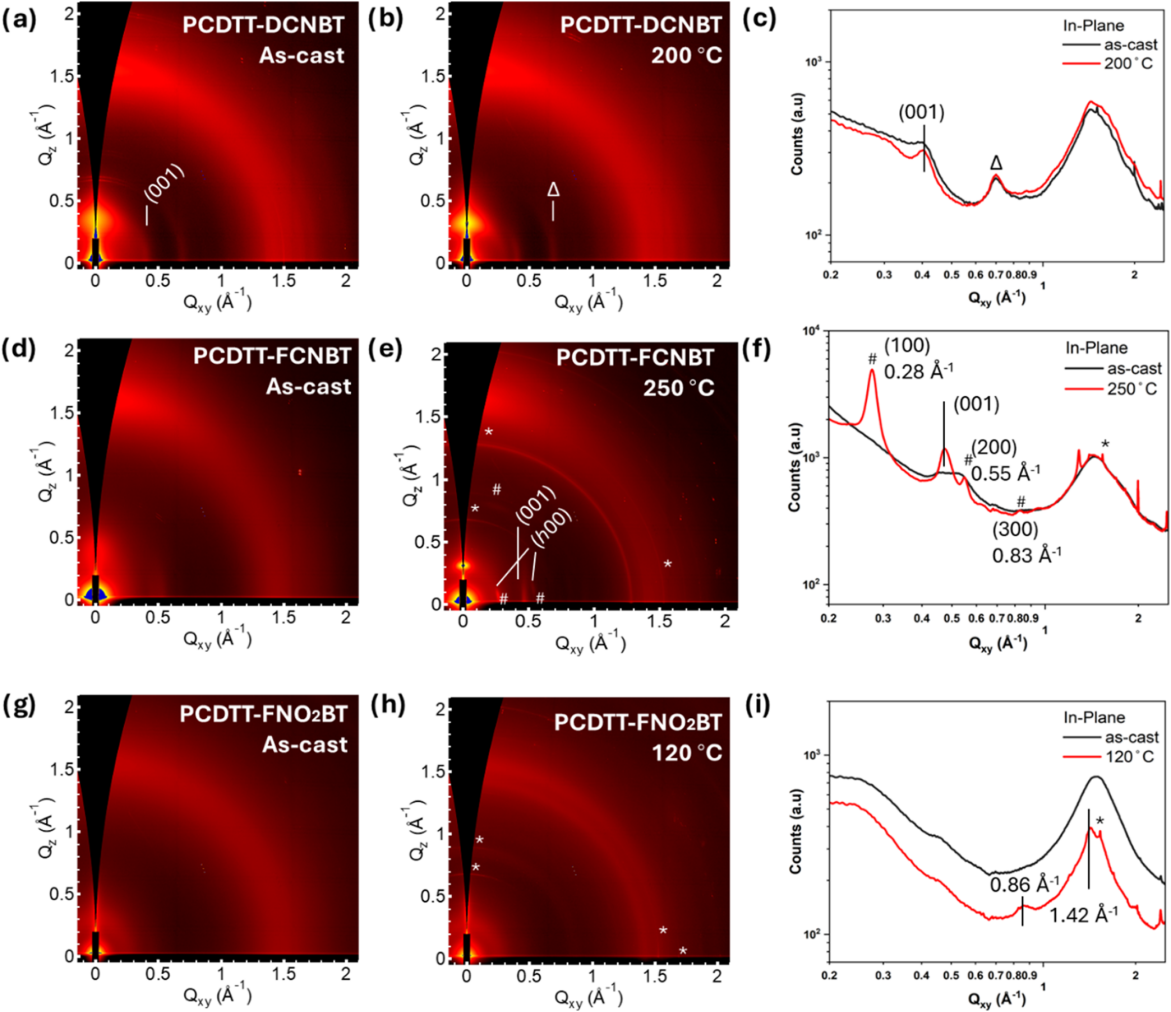
(a, b, d, e, g, h) 2D GIWAXS patterns and (c, f, i) scattering profiles of in-plane for as-cast and annealed PCDTT-DCNBT (top row), PCDTT-FCNBT (middle row), and PCDTT-NO_2_FBT (bottom row). For PCDTT-DCNBT, indexing of the IP peak at 0.7 Å^–1^ (marked Δ) is uncertain, possibly the (200). Peaks marked with * are artifacts and originate from the substrate.

The effect of temperature in polymer films was investigated using grazing-incidence wide-angle X-ray scattering (GIWAXS) analysis (Figure 3) and temperature UV–vis (Figure S12). We note that for all polymers, no obvious transitions were observed by differential scanning calorimetry (DSC) (Figure S13), as is often observed for conjugated donor–acceptor-type polymers.

GIWAXS reveals that the as-cast polymers were relatively disordered, especially when compared to polymers such as P3HT or PBTTT. The scattering pattern of PCDTT-DCNBT (Figure 3a,b) resembles that of IDT-BT^46^ with coexisting face-on and edge-on oriented crystallites evidenced by the coappearance of side chain stacking and backbone stacking peaks in the out-of-plane direction. The in-plane diffraction peaks of PCDTT-DCNBT are assigned to backbone reflections, i.e., (00*l*) with a backbone repeat distance of *d*_001_ = 15.3 Å based on the location of the (001) peak at *q*_001_ = 0.41 Å^–1^. The π -stacking distance for PCDTT-DCNBT is also determined to be *d*_010_ = 4.0 Å. Annealing PCDTT-DCNBT at 200 °C results in a narrowing of the in-plane (IP) backbone peak, indicating an increase in coherence length, while no significant change to the OOP π–π stacking peak is observed. This slight improvement in crystallinity correlates with the enhanced electron mobility after annealing at this temperature. The increase in the coherence length also correlates with the increase in UV/vis absorption observed after annealing at this temperature (Figure S12).

The as-cast GIWAXS pattern of PCDTT-FCNBT (Figure 3d) shows similar but weaker scattering features compared to those of as-cast PCDTT-DCNBT. The first-order backbone peak appears at a higher *q* value, *q*_001_ = 0.46 Å^–1^, indicating a smaller *d*-spacing along the backbone direction of *d*_001_ = 13.7 Å. This observation suggests a different backbone conformation and/or different tilting of the backbone along this crystallographic axis. A slightly smaller π-stacking distance is also observed for PCDTT-FCNBT at *d*_010_ = 3.9 Å. Similar to PCDTT-DCNBT, annealing PCDTT-FCNBT at 180 °C promotes lamellar and backbone order as indicated by the stronger OOP (100) and IP (001) peaks relative to the OOP π–π (010) peak, along with a larger coherence length as indicated by peak narrowing (Figure S14a). This increase in order coincides with the first peak in electron mobility following the annealing of the device. Annealing at a higher temperature of 250 °C promotes crystallization of PCDTT-FCNBT into a crystalline form with a nonorthorhombic unit cell as indicated by the off-axis peaks (marked #, Figure 3e) including the near IP (*h*00) peaks. This improved crystallinity correlates with the maximum mobility achieved for PCDTT-FCNBT. The absorption of the film decreases after annealing at this temperature and subtly changes shape (Figure S12), which we relate to the different orientations with respect to the substrate and changes in the electronic couplings between the chains.

Lastly, as-cast PCDTT-NO_2_FBT appears to be essentially amorphous with a lack of well-defined scattering features (Figure 3g). When PCDTT-NO_2_FBT is annealed at 120 °C, two isotropic rings form at 0.86 and 1.42 Å^–1^, Figure 3h, correlating with the optimized mobility of this polymer. These isotropic rings then disappear upon annealing at 200 °C (Figure S14b), at which the transistor performance also drops significantly. Notably, unlike the two other polymers, PCDTT-NO_2_FBT does not exhibit (00*l*) backbone reflections, indicating reduced backbone ordering. Polymer PCDTT-NO_2_FBT shows similar absorption in the as-cast and 120 °C annealed films. When the annealing temperature is increased to 200 °C, a blue shift and decrease in absorption intensity is observed.

Overall, considering the changes in the nature of the BT group on the transistor performance, we show that the lower electron-withdrawing strength of fluorine is compensated by its relatively smaller size and its preference for the anti-conformation with the adjacent CDTT, resulting in a more linear backbone compared to the dicyanated monomer. Together with improvement in backbone planarity, this correlates with improved crystallinity after annealing, resulting in the highest electron mobility of PCDTT-FCNBT from the three polymers. We note that the polymer molecular weight may also play a role in the observed differences in device performance, considering the higher values of PCDTT-FCNBT compared to PCDTT-DCNBT.^15^ Comparing the cyano to nitro group, we show that the latter is less effective as a strong electron-withdrawing group since its larger size results in twist away from coplanarity with the backbone, thereby reducing the overlap of the nitro group’s π system and reducing its resonance-withdrawing effects. The larger size also results in more pronounced deviations from backbone coplanarity, and the combined effect results in the lowest electron mobility of the three polymers.

## Conclusions

In summary, we have presented the synthesis and characterization of three novel D–A polymers that share the same CDTT donor unit but differ in the functionalization of the benzothiadiazole acceptor unit. The most electron-deficient dicyanated monomer DCNBT resulted in polymer PCDTT-DCNBT that was compared and contrasted with its singly fluorinated analogue PCDTT-FCNBT. Substitution of one cyano group with one fluorine atom results in a blue shift in solution absorption, but interestingly, both polymers exhibit the same absorption maximum in the solid state. When the two materials were implemented in OFET devices, the more electron-deficient PCDTT-DCNBT polymer showed poor device performance with an electron mobility at μ_e,max_ ≈ 0.031 cm^2^ V^–1^ s^–1^, whereas the polymer PCDTT-FCNBT exhibited significantly improved performance, with an electron mobility 13 times higher at μ_e,max_ ≈ 0.4 cm^2^ V^–1^ s^–1^ after annealing. The presence of fluorine promotes backbone planarity and changes the lowest energy conformation from *syn* to *anti*, resulting in a more linear backbone that exhibits high degrees of crystallinity. Additionally, we report the first nitro group functionalization of a BT unit in a polymeric semiconductor. The replacement of one cyano group with one nitro group produced polymer PCDTT-NO_2_FBT, and resulted in a blue shift of absorption and a 0.2 eV increase in the band gap. The effectiveness of the nitro group as an electron-withdrawing group was reduced by steric effects, which twisted the nitro group out of conjugation with benzothiadiazole, limiting its role as a π-acceptor and resulting in a reduced transistor performance with μ_e,max_ ≈ 0.024 cm^2^ V^–1^ s^–1^ when compared to its cyano counterpart (μ_e,max_ ≈ 0.031 cm^2^ V^–1^ s^–1^).

Overall, we have shown that the systematic tuning of cyano and fluorine groups is an effective method for tuning the morphology and electron transport properties. The presence of fluorine is beneficial for enhancing polymer electron mobility by improving backbone linearity, planarity, and crystallinity. Nitro groups have not been greatly investigated as π-acceptors, and in this study, we demonstrate their poor efficacy as a strong electron-withdrawing group. Future use of the nitro group requires careful consideration of their sterics, such that the nitro group is able to orient in the same plane as the conjugated backbone, allowing for full overlap of the respective π systems and maximization of their undoubted electron-accepting behavior.

## Supporting Information Available

Experimental section; characterization techniques; device fabrication; ^1^H, ^19^F, and ^13^C NMR spectra of intermediates, monomers and polymers; mass spectra; FTIR spectra; theoretical calculations; transfer and output plots; cyclic voltagramms; PESA plots; UV–vis spectra of annealed films; and TGA, DSC, and GIWAXS plots (PDF)
