# Supplementary material for: Selective Tuning of Benzothiadiazole Functionality Enables High Crystallinity and Mobility in Regiorandom n-Type Polymers for Organic Field-Effect Transistors
Source: Macromolecules. 2025 Mar 21;58(7):3694–703. doi: 10.1021/acs.macromol.4c02854 (PMC11984307; doi:10.1021/acs.macromol.4c02854)
Supplement: Supplementary file 1 — ma4c02854_si_001.pdf [file ma4c02854_si_001.pdf]

Supporting Information:
Selective tuning of benzothiadiazole functionality enables high crystallinity and mobility in regiorandom n-type polymers for organic field effect transistors

Panagiota Kafourou,[1,2] Qiao He,[2] Xiantao Hu,[2] Mohamad Insan Nugraha,[1,4,5] Wen Liang Tan,[3] Joel Luke,[1] Bowen Ding,[2] Christopher R. McNeill,[3] Thomas D. Anthopoulos,[1,6] Martin Heeney[1]

[1]King Abdullah University of Science and Technology (KAUST), KAUST Solar Center, Physical Sciences and Engineering Division (PSE); Thuwal 23955-6900, Kingdom of Saudi Arabia.

[2]Department of Chemistry and Centre for Processable Electronics, Imperial College London, London W12 0BZ, UK.

[3]Department of Materials Engineering Monash University Clayton, Victoria 3800, Australia

[4]Research Center for Nanotechnology Systems, National Research and Innovation Agency (BRIN), South Tangerang, Banten 15314, Indonesia

[5]Collaboration Research Center for Advanced Energy Materials, National Research and Innovation Agency – Institut Teknologi Bandung, Jl Ganesha 10, Bandung, 40132, Indonesia

[6]Henry Royce Institute and Photon Science Institute, Department of Electrical and Electronic Engineering, The University of Manchester, Oxford Road, Manchester, M13 9PL, United Kingdom

Contents

1. Methods 2
2. Electrochemical and Thermal characterisation 2
3. Density functional theory (DFT) calculations 2
4. OFET fabrication 3
5. Grazing-Incidence Wide-Angle X-ray Scattering (GIWAXS) 3
6. Synthetic procedures 3
8. Conformation calculations 7
9. OFET Characteristics 10
10. Morphology 16
11. NMR and Mass Spectra 16
12. References 16

1. Methods

Nuclear magnetic resonance (NMR) spectra were recorded on Bruker AV-400 (400 MHz) spectrometers in $CDCl_3$ using the residual solvent resonance of o-DCB-d4, or D_2-1,1,2,2-tetrachloroethane. Polymer films were prepared at 5 mg/ml. Polymer PCDTT-DCNBT was dissolved in *o*-dichlorobenzene, polymer PCDTT-FCNBT in chlorobenzene and PCDTT-NO_2FBT in chloroform overnight. UV-Vis spectra in chloroform solution (PCDTT-NO_2FBT) were recorded in a UV-1601 Shimadzu UV-vis spectrometer. UV-Vis-NIR spectra were recorded in a Cary 7000 UV-Vis-NIR Universal Measurement Spectrophotometer.

2. Electrochemical and Thermal characterisation

Cyclic voltammograms were recorded using a Metrohm Autolab PGSTAT101 potentiostat/galvanostat. The experimental setup consisted of an Ag/Ag+ reference electrode, a platinum wire counter electrode and a platinum working electrode, and all measurements were carried out under nitrogen at room temperature. Measurements were performed in anhydrous, degassed solutions CH_3CN with tetrabutylammonium hexafluorophosphate (0.1 M) electrolyte. Polymer thin films were prepared via dropcasting from CB solution on platinum working electrode. After each measurement, an arbitrary amount of ferrocene was added to the solution as an internal reference. The potentials were referenced to those of ferrocene when a ferrocene/ferrocenium reference redox system of 4.8 eV below the vacuum level was used as an internal standard, and the conversion from electrochemical potentials to electron volts was done using the formula $E(eV) = -E_{redox} - 4.8$ eV.[1, 2] Any solvent effects were neglected.

Photoelectron spectroscopy in air measurements were recorded with a Riken Keiki AC-2 PESA spectrometer with a power setting of 15 nW and a power number of 0.3. Thermal gravimetric analysis plots were obtained with a PerkinElmer Pyris 1 TGA machine at a scan rate of 10 °C min^{-1}, under a nitrogen atmosphere.

Differential scanning calorimetry experiments were carried out with a TA Instruments DSC TZero Q20 v24.10 instrument at a scan rate of 10 °C min^{-1} and analysed using TA Instruments Universal Analysis 2000 v4.5A software.

3. Density functional theory (DFT) calculations

DFT calculations were conducted using Gaussian 09 software on the Imperial College High-Performance Computing Service.[3] All simulations were carried out on single molecules in the gas phase at the B3LYP level of theory with the basis set 6-31G(d,p).[4-6] Alkyl side chains were replaced by methyl groups to reduce the computation time. Structures were optimized to a local minimum energy conformation, and frozen dihedral angles were used to simulate molecular conformational changes.

4. OFET fabrication

Polymer PCDTT-DCNBT was dissolved in *o*-dichlorobenzene, polymer PCDTT-FCNBT in chlorobenzene and PCDTT-NO_2FBT in chloroform overnight. Polymer PCDTT-DCNBT was spin coated at 2000 rpm for 60 seconds. Polymers PCDTT-FCNBT and PCDTT-NO_2FBT were spin coated at 1000 rpm for 60 seconds. The concentration of all solutions was 5 mg / mL. All films were prepared under inert atmosphere. Polymers were annealed at the indicated temperature for 30 mins before the deposition of the gate dielectric. Top gate/bottom contact (TG/BC) devices were fabricated on glass substrates using Al/Au (5/45 nm) source–drain electrodes, polymethylmethacrylate (PMMA) in butyl acetate (80 mg / mL, 2000 rpm, 60s) dielectric, and Al (70 nm) gate electrode. PMMA thickness was 700 nm. Thermal annealing of PMMA was at 90 °C for 2 hours. The electron mobility in the devices was calculated using equation in the saturation regime: $\mu_{sat}=(2L/WC_i)(dI_{DS}^{1/2}/dV_G)^2$, where I_D is the source–drain current, C_i (measured capacitance, 3.15 nF cm^{-2}) is the capacitance per unit area, L is the channel length, *W* is the channel width, and V_G is the gate voltage.

5. Grazing-Incidence Wide-Angle X-ray Scattering (GIWAXS)

Polymer PCDTT-DCNBT was dissolved in *o*-dichlorobenzene, polymer PCDTT-FCNBT in chlorobenzene and PCDTT-NO_2FBT in chloroform overnight. Samples were prepared by spin coating from solution (5 mg / mL). Polymer PCDTT-DCNBT was spin coated at 2000 rpm for 60 seconds. Polymers PCDTT-FCNBT and PCDTT-NO_2FBT were spin coated at 1000 rpm for 60 seconds. Thermal annealing at different temperatures (120, 180, 200, 250 °C) for 30 min. GIWAXS measurements were performed at the SAXS/WAXS beamline at the Australian Synchrotron. [7] Two-dimensional scattering patterns were recorded on a Dectris Pilatus 2M detector with 15 keV photons used to probe the samples. The samples were measured in vacuum, with the entire beam path in vacuum to minimise background scatter. The total exposure time was 3 s, with the reported images a composite of three separate 1 s exposures taken with different lateral detector offsets to fill in the regions missed by gaps in the detector. A silver behenate standard was used to calibrate the sample-to-detector distance. Results were analysed using NIKA 2D[8] implemented in IgorPro.

6. Synthetic procedures

Synthesis of **1**:[9, 10] To a nitrogen flashed flask, a solution of 4,5-diaminopthalonitrile (3 g, 19.0 mmol) in methanol (350 mL) was added, followed by potassium bromide (30 g, 250 mmol). The suspension was cooled to 0 °C followed by dropwise addition of hydrobromic acid (62 wt%, 3.8 g, 47.5 mmol) and *tert*-butylhydroperoxide (70 wt%, 6.9 g, 76 mmol). The suspension was left to gradually warm up to r.t and was monitored by TLC [eluent: ethyl acetate:pet.ether

(5:1)]. After 6 hours of stirring, *tert*-butylhydroperoxide (6 mL) was added and monitored until reaction completion. The reaction mixture was then filtered and washed with methanol (2 x 50 mL). In a clean Büchner flask, the solid residue was washed with hot ethyl acetate (3 x 150 mL) and the combined ethyl acetate solutions were concentrated to afford the product as a purple solid (4.6 g, 14.9 mmol, 78%). ^{1}H NMR (400 MHz, DMSO-d$_6$): δ 6.40 (s, 4H) ppm; ^{13}C NMR (101 MHz, DMSO-d$_6$): δ 136.9, 116.3, 105.98, 105.95 ppm; HRMS (M+, EI): *m/z* calcd. for $C_8H_4Br_2N_4$: 314.8875, found: 314.8873.

4,7-Dibromo-5,6-dicyanobenzo[*c*][1,2,5]thiadiazole (**DCNBTBr$_2$)**. Thionyl chloride (3.0 g, 25.4 mmol) was added dropwise to a solution of compound **1** (4.0 g, 12.7 mmol) in a mixture of $CHCl_3$ (100 mL) and triethyl amine (5.1 g, 50.8 mmol) at 0 °C. After addition, the mixture was refluxed for 12 hours, and allowed to cool to r.t. After the addition of water (150 mL) the solution was extracted with CH_2Cl_2 (2 x 100 mL). The combined organics were further washed with water (100 mL) and brine (100 mL) and finally dried over $MgSO_4$. The solvent removed under reduced pressure and the crude product was purified by column chromatography [eluent: pet. ether/ CH_2Cl_2, 1:5 (v:v)]. The pure product was collected and triturated with heptane for 16 hours and filtered to afford the product as white solid (3.0 g, 8.7 mmol, 68%). m.p. (DSC) 298.4 °C.^{13}C NMR (101 MHz, DMSO-d$_6$): δ 152.5, 122.9, 116.1, 114.7 ppm; HRMS (M+, EI): *m/z* calcd. for $C_8N_4Br_2S$: 341.8216, found: 343.8220.

4,7-Dibromo-6-fluoro-5-cyanobenzo[*c*][1,2,5]thiadiazole (**FCNBTBr$_2$**). A solution of compound 2 (150 mg, 0.45 mmol), KCN (29.1 mg, 0.45 mmol) and 18-crown-6 (12.02 mg, 0.05 mmol) in a mixture of anhydrous THF (15 mL)/DMF (4 mL) was heated at 60 ℃ to reflux under N_2 for 16 h. After cooling to room temperature, the reaction mixture was diluted with water, extracted with DCM, washed with brine and dried with magnesium sulfate. After removing the solvent, the crude product was further purified using silica gel column chromatography with the mixture of petroleum ether and dichloromethane (3:1, v/v) as the eluent, yielding starting materials (114 mg, 76%, R_f 0.7) and the product solid (15 mg, 10%, R_f 0.6). ^{13}C NMR (101 MHz, $CDCl_3$) δ 159.70, 157.11, 153.47, 153.40, 149.57, 121.65, 121.61, 111.64, 110.20, 109.95, 100.12, 99.88 ppm. ^{19}F NMR (377 MHz, $CDCl_3$) δ -98.26 ppm. MS (*m/z*): [M^+] calcd. for $C_7Br_2FN_3S$: 336.96, Found: 336.8143 (ESI). FTIR (ν cm^{-1}): 2235.5 [ν(CN) stretching].

4,7-Dibromo-5-fluoro-6-nitrobenzo[*c*][1,2,5]thiadiazole (**NO_2FBTBr$_2$**). To a solution of CF_3SO_3H (1.69 g ml^{-1}, 5.38 ml) at 0 ℃ was added fuming HNO_3 (1.42 g ml^{-1}, 1.95 ml) dropwise. Compound 3 (1.55 g, 4.9 mmol) was then added, and the reaction mixture was stirred at 50 ℃ under nitrogen for 16 h. The crude product was poured into iced water, filtered to get solid, washed with water, yielding solid (1.09 g, 61%). ^{13}C NMR (101 MHz, $CDCl_3$) δ 153.33, 151.61, 151.56, 150.74, 148.83, 107.50, 100.98, 100.75 ppm. ^{19}F NMR (377 MHz, DMSO) δ -116.07 ppm. MS (*m/z*): [M^+] calcd. for $C_6Br_2FN_3O_2S$: 356.95, Found: 356.8046 (ESI).

General method for polymerisation: A 2 mL microwave vial was dried and purged with nitrogen. Compound 4 (100 mg, 0.094 mmol), **BT-monomer** (0.094 mmol), tris(dibenzylideneacetone) dipalladium(0)-chloroform adduct (1.9 mg, 0.0019 mmol), tris(o-tolyl) phosphine (2.3 mg, 0.0075 mmol) and the vial was sealed and purged with nitrogen for 10 minutes. Degassed toluene (1 mL) was added and the mixture was further degassed for 3 minutes. The vial was placed in a microwave reactor and heated to 120 °C for 2 min, 140 °C for 2 min, 160 °C for 2 min and 180 °C for 40 min. After cooling to r.t, the product was precipitated in MeOH and filtered into a thimble. Soxhlet extraction was carried out with MeOH, acetone, hexane and chloroform.

Synthesis of polymer **PCDTT-DCNBT**. The polymer was soluble in chlorobenzene (67 mg). (67 mg, 0.070 mmol, 75 %). Mn = 20.1 kDa, Mw = 29.0 kDa, Mw/Mn (Đ) = 1.4; ^1H NMR (400 MHz, 373 K, TCE-d_2) δ: 8.79 (s, 2H), 1.36-1.23 (m, 60 H), 0.95-0.91 (m, 6 H) ppm.

Synthesis of polymer **PCDTT-FCNBT**. The polymer was soluble in chlorobenzene (50 mg). (50 mg, 0.053 mmol, 56%). Mn = 56.9 kDa, Mw = 91.8. kDa, Mw/Mn, (Đ) = 1.6; ^1H NMR (500 MHz, 373 K, TCE-d_2): δ 8.88 (d, J = 5 Hz, 2H), 8.80-8.78 (m, 2H), 1.34 - 1.22 (m, 60H), 0.93 (t, J = 5 Hz, 6H) ppm; ^{19}F NMR (470 MHz, 373 K, TCE-d_2): δ 103.82, 104.04 ppm.

Synthesis of polymer **PCDTT-NO_2FBT**. Polymer was soluble in chloroform solution (65 mg). (65 mg, 0.067 mmol, 72%). Mn = 33.9 kDa, Mw = 69.3. kDa, Mw/Mn (Đ) = 2.0; ^1H NMR (500 MHz, 373 K, TCE-d_2): δ 8.80 (s, 2H), 8.06 (s, 2H), 1.48-1.14 (m, 60H), 0.93 (t, J = 5 Hz, 6H) ppm; ^{19}F NMR (470 MHz, 373 K, TCE-d_2): δ 121.55, 161.62 ppm.

7. Optoelectronic and Thermal Properties

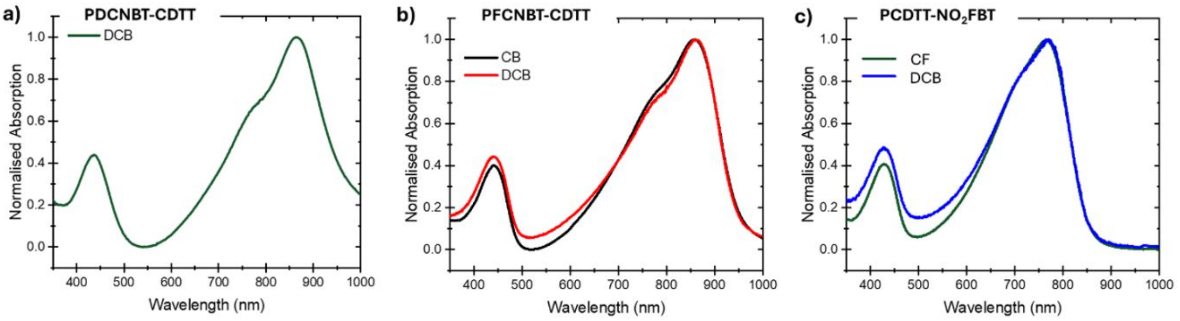

Figure S1. Impact of spin-coating from different solvents UV-Vis absorption (1,2-dichlorobenzene (DCB), chlorobenzene (CB) and chloroform (CF).

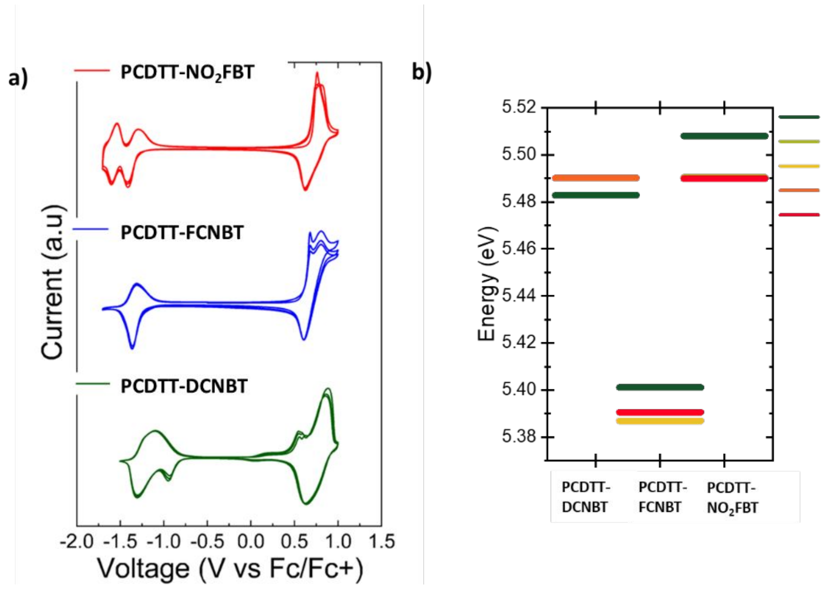

Figure S2. a) CV of drop cast samples in acetonitrile-[n-Bu_4N]PF_6 solution (0.1 M) at 100 mVs^{-1} scan rate b) PESA of polymers in thin films at different annealing temperatures. Films prepared from **PDCNBT-CDTT**, **PFCNBT-CDTT** and **PNO_2FBT-CDTT** films were prepared in *o*-dichlorobenzene (DCB), chlorobenzene (CB) and chloroform (CF).

8. Conformation calculations

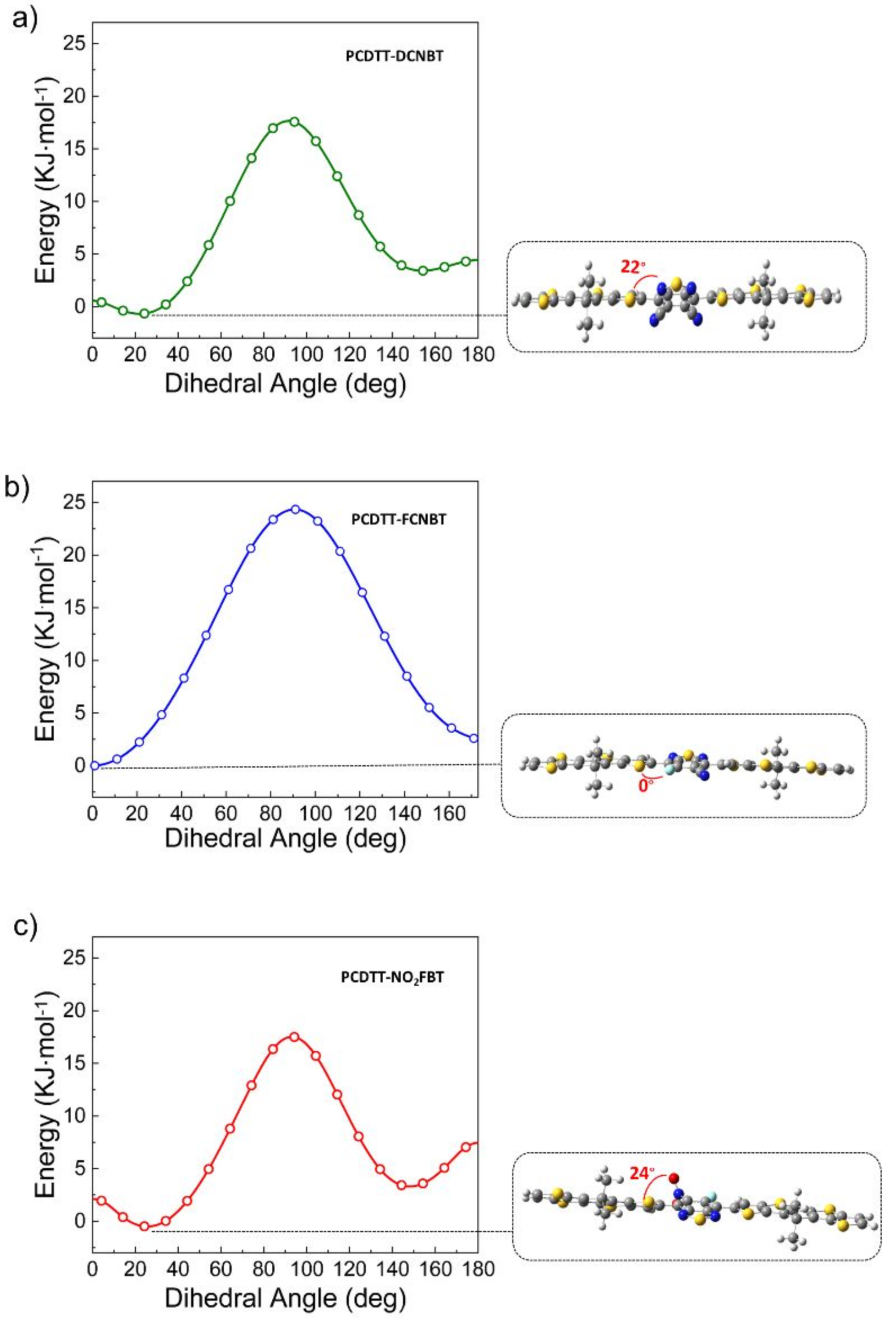

Figure S3. Relaxed potential energy scans (PES). The angles between the BT and CDTT units were scanned between 0-180 degrees, indicating the global minimum energy conformation a) bond rotation between DCNBT and CDTT, 0° corresponds to the *syn* confomer b) bond rotation between FCNBT and CDTT, 0° corresponds to the *anti* confomer and c) bond rotation between NO_2FBT and CDTT, 0° corresponds to the *syn* confomer.

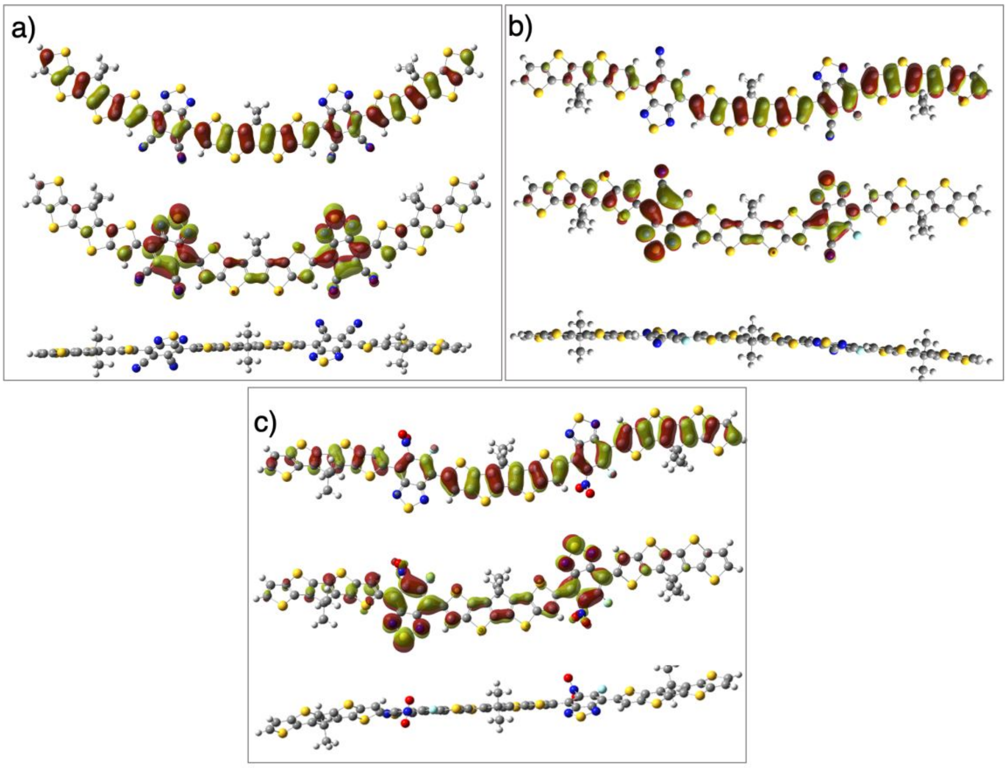

Figure S4. Orbital visualizations of the minimized energy structures and side view of polymers a) **PCDTT-DCNBT**, b) **PCDTT-FCNBT** and c) **PCDTT-NO_2FBT**. In each picture is shown; HOMO (top), LUMO (middle) and side view (bottom).

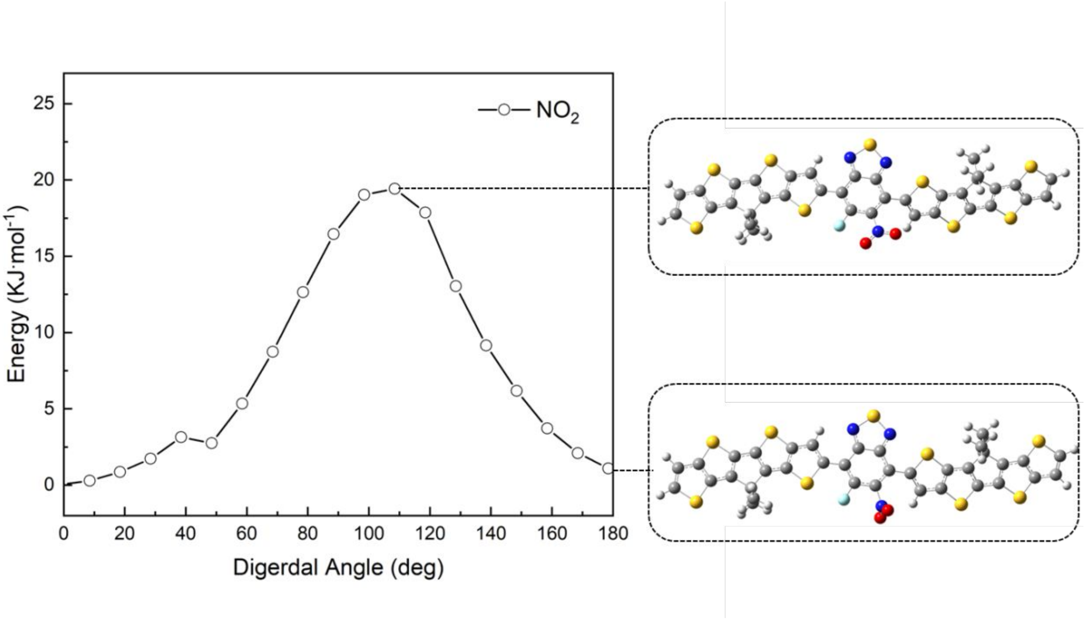

Figure S5. PES scan of the NO_2 group indicating minimum and maximum and minimum energy conformation. At minimum energy NO_2 group is roughly perpendicular to the backbone conjugation and at maximum energy, NO_2 group is in the same plane as the backbone conjugation.

9. OFET Characteristics

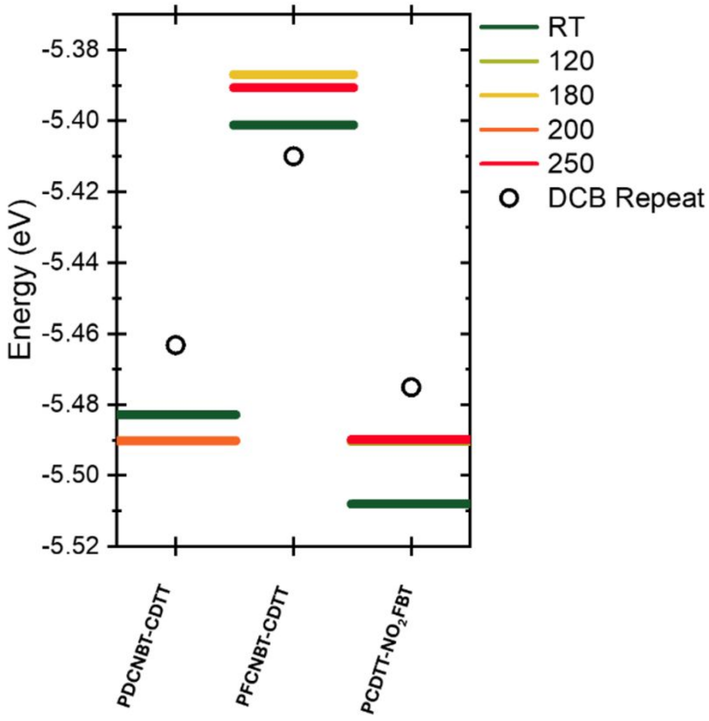

Figure S6. PESA measurements from figure S2, and repeat measurements for films prepared from DCB at RT. Differences in energy are within error, and trends in film annealing temperature remain the same.

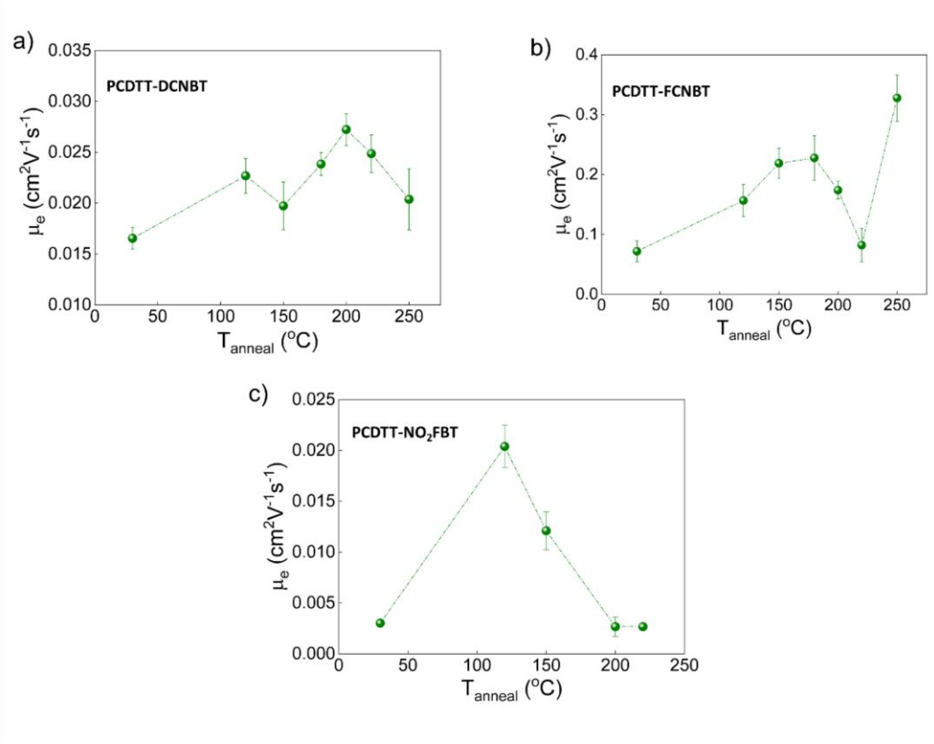

Figure S7. Field effect mobility as a function of annealing temperature for polymers a) **PCDTT-DCNBT**, b) **PCDTT-FCNBT** and c) **PCDTT-NO$_2$FBT**.

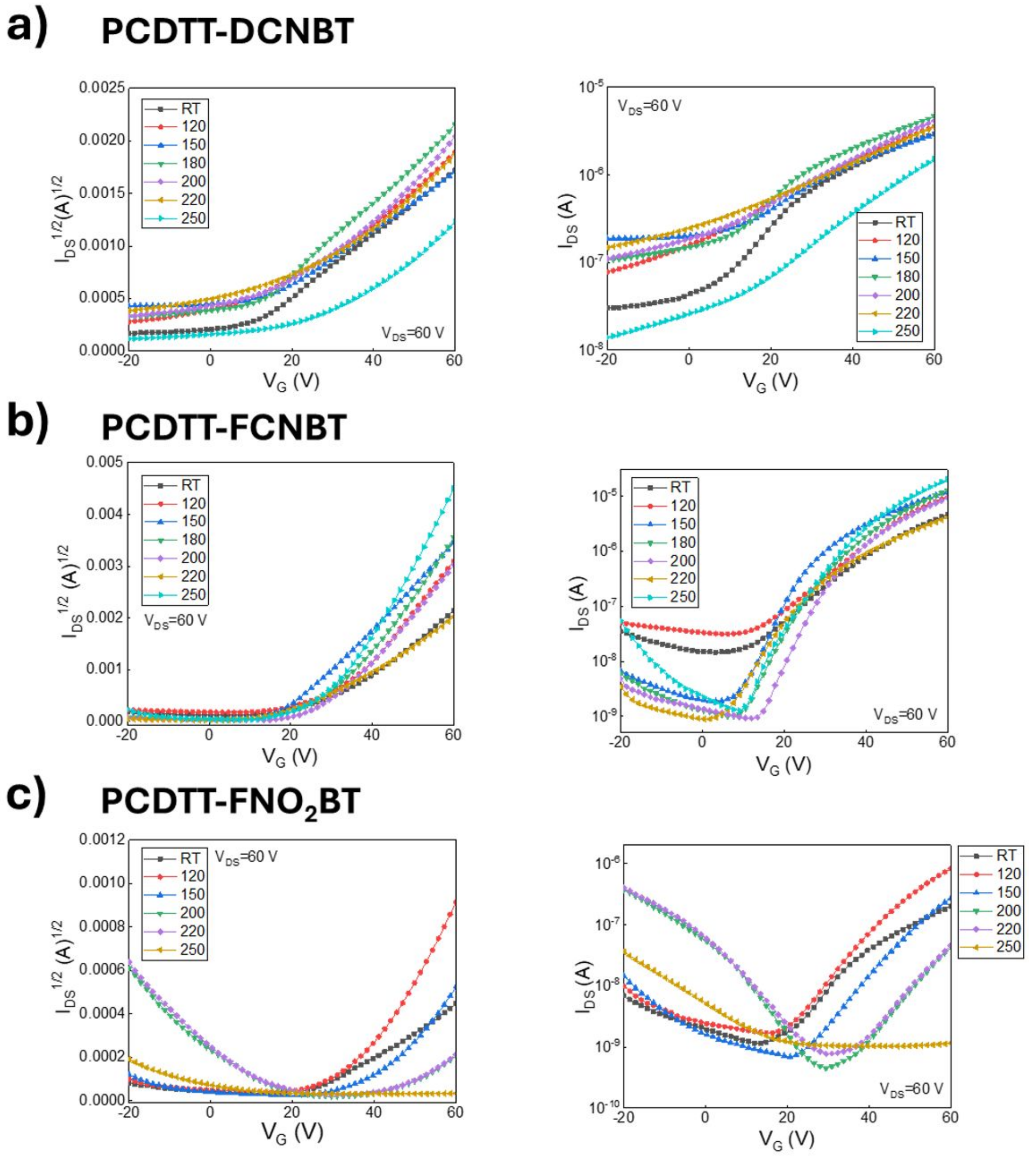

Figure S8. Transistor transfer curves at different annealing temperatures.

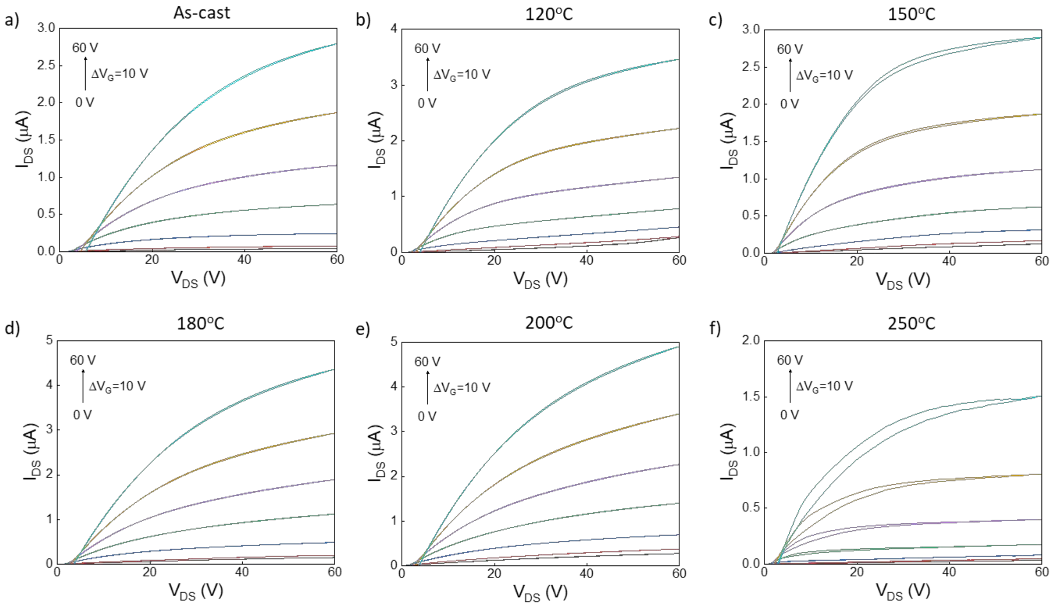

Figure S9. **PDCNBT-CDTT** output curves at different annealing temperatures

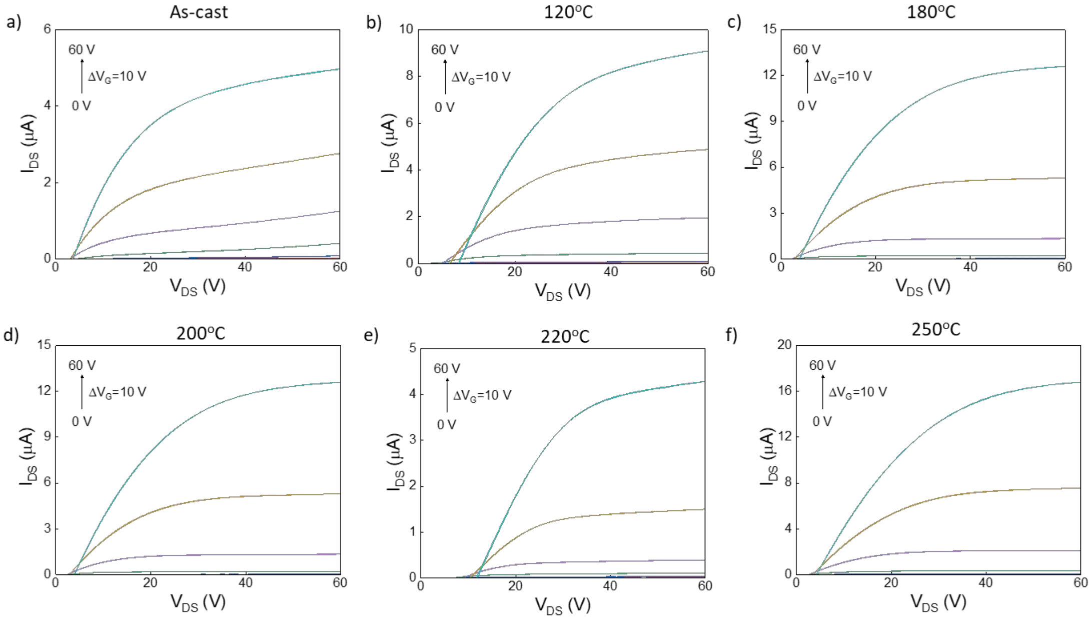

Figure S10. **PFCNBT-CDTT** output curves at different annealing temperatures

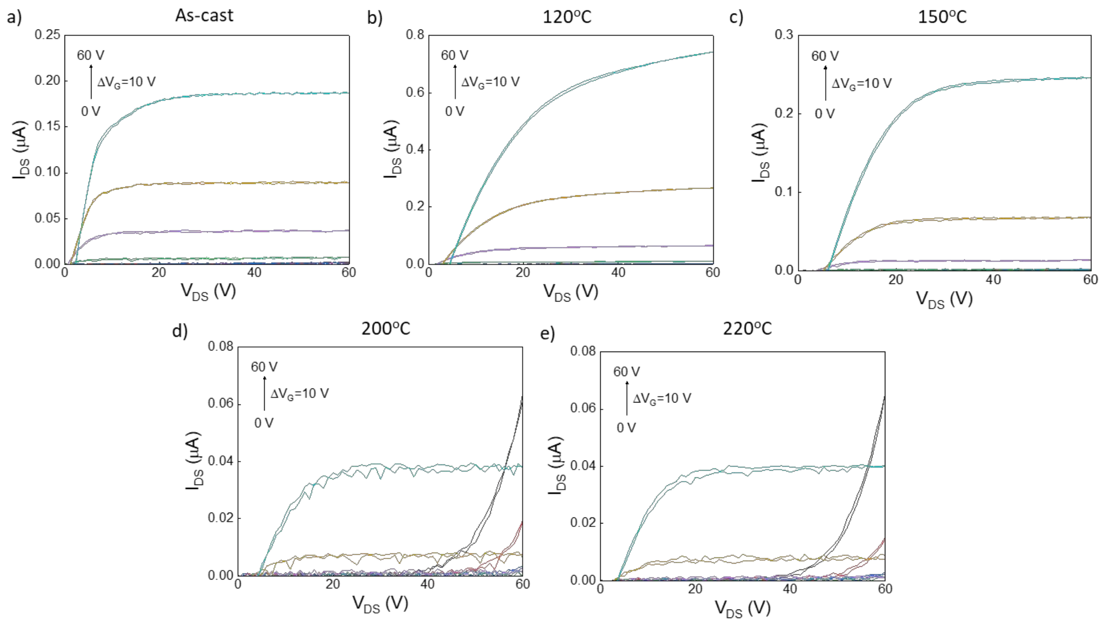

Figure S11. **PNO_2FBT-CDTT** output curves at different annealing temperatures

Table S1. Polymer mobilities in relation to annealing temperature

Polymers	T_{anneal} (^oC)	μ_{avg} ($cm^2V^{-1}s^{-1}$)	μ_{max} ($cm^2V^{-1}s^{-1}$)
**PCDTT-DCNBT**	As-cast	0.016	0.018
	120	0.023	0.024
	150	0.02	0.024
	180	0.024	0.024
	**200**	**0.027**	**0.031**
	220	0.025	0.027
	250	0.02	0.035
**PCDTT-FCNBT**	As-cast	0.072	0.095
	120	0.16	0.2
	150	0.22	0.26
	180	0.24	0.3
	200	0.17	0.19
	220	0.1	0.12
	**250**	**0.33**	**0.4**
**PCDTT-NO_2FBT**	As-cast	3×10^{-3}	3.2×10^{-3}
	120	0.02	0.024
	150	0.012	0.015
	200	2.7×10^{-3}	3.8×10^{-3}
	220	2.7×10^{-3}	3.1×10^{-3}
	250	-	

10. Morphology

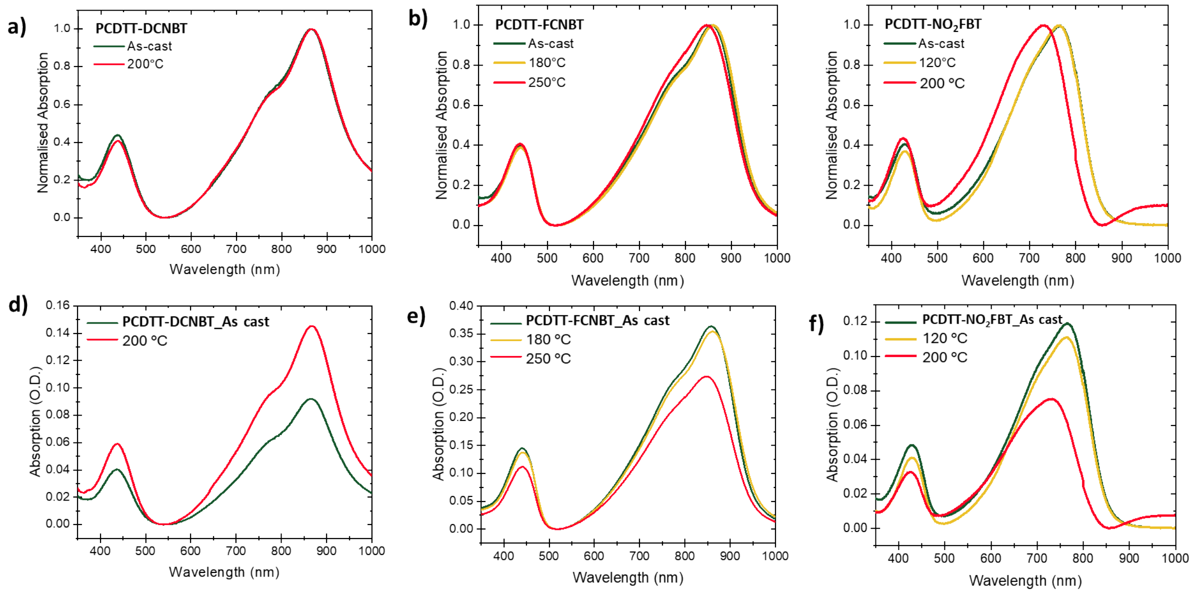

Figure S12. UV-Vis spectra after different annealing temperatures (a-c normalised; d-f non-normalised plots). Polymer PCDTT-DCNBT was dissolved in *o*-dichlorobenzene, polymer PCDTT-FCNBT in chlorobenzene and PCDTT-NO_2FBT in chloroform overnight. Dynamic spin-coating was used for film preparation.

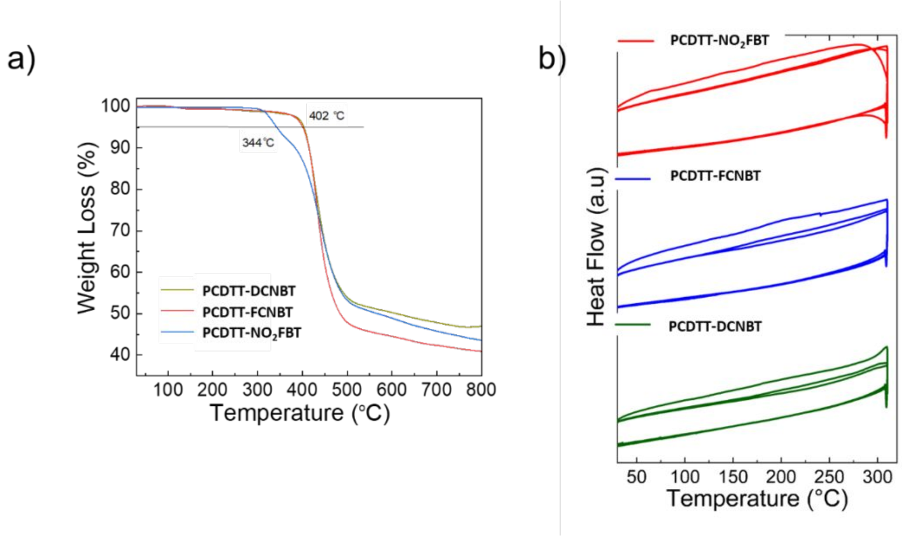

Figure S13. a) TGA conditions; 25.0.- 800.0 °C, 10.00 K/min, N2 50.0 ml/min, indicating degradation temperature for each polymer. B) DSC conditions at 30-320 °C, rate 10 °C/min, in N_2.

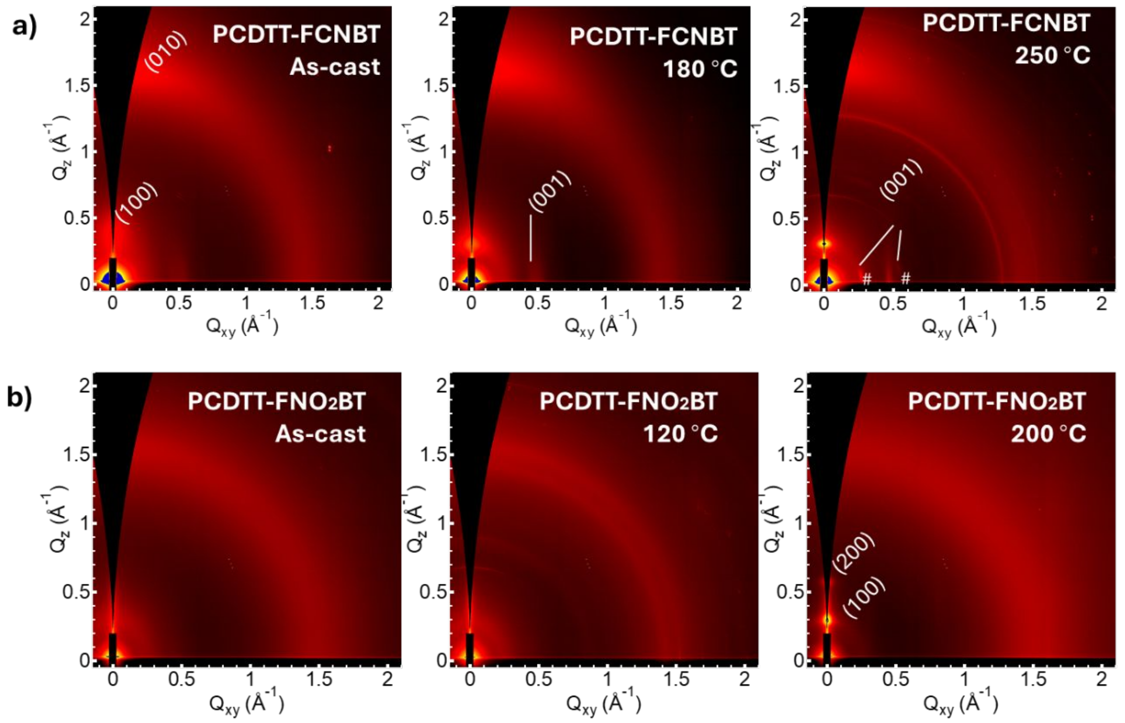

Figure S14. 2D GIWAXS patterns of PCDTT-FCNBT and PCDTT-NO_2FBT, as-cast and at different annealing temperatures.

11. NMR and Mass Spectra

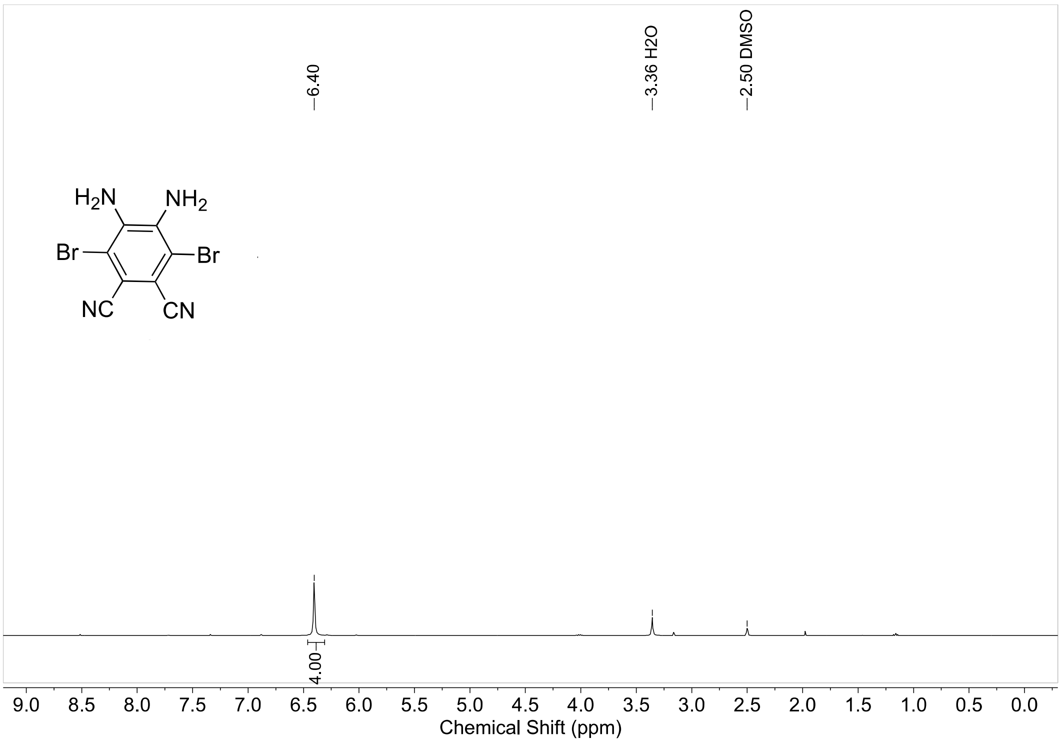

Figure S15. ^{1}H NMR spectrum of compound **1** in DMSO.

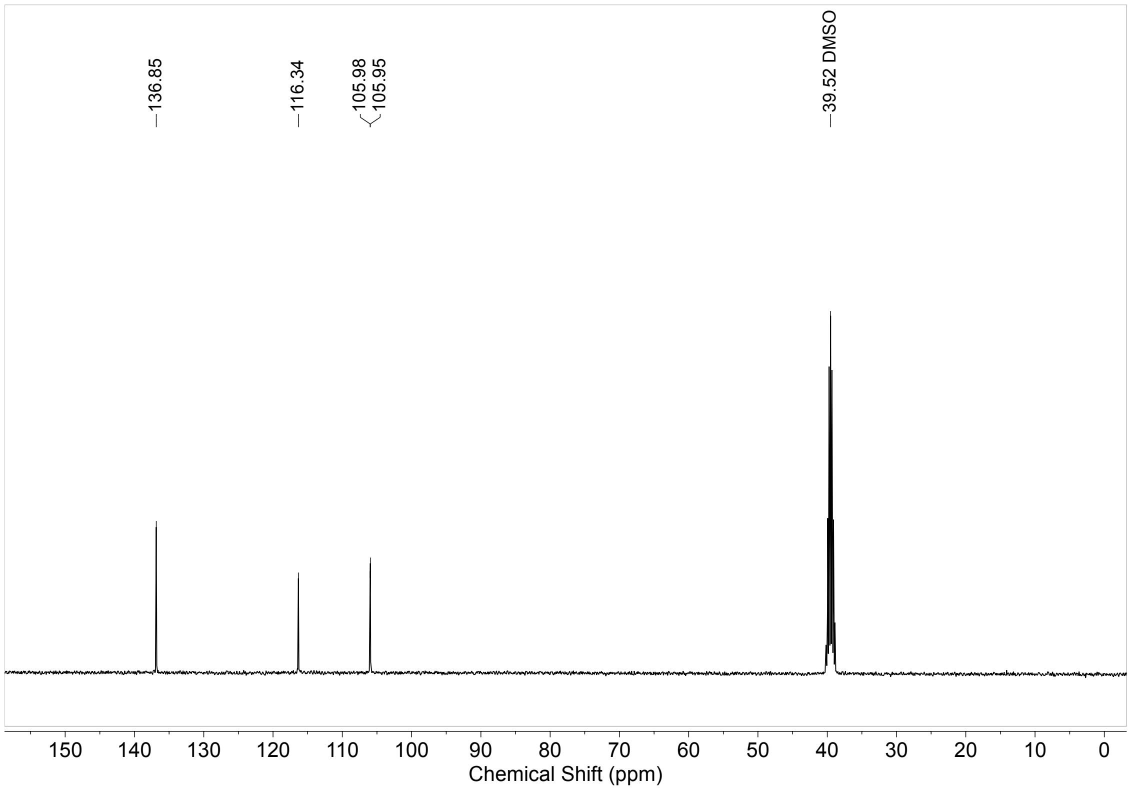

Figure S16. ^{13}C NMR spectrum of compound **1** in DMSO.

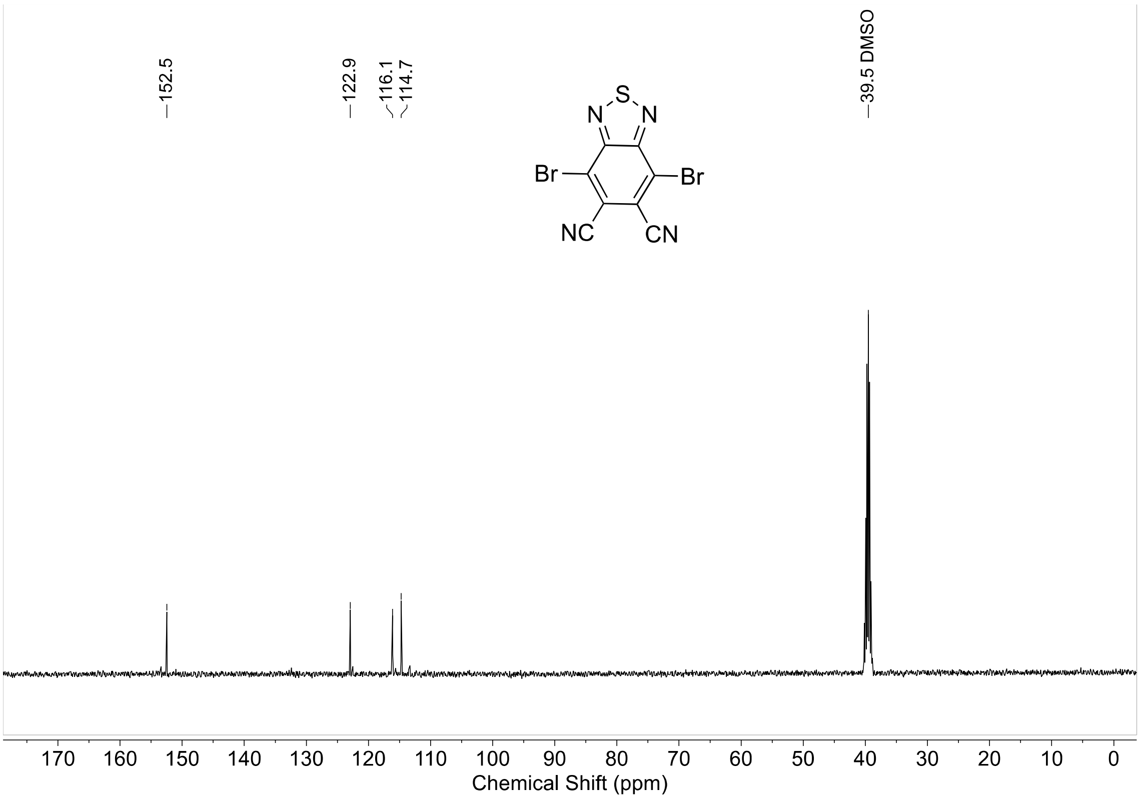

Figure S17. ^{13}C NMR spectrum of monomer **DCNBTBr$_2$** in DMSO.

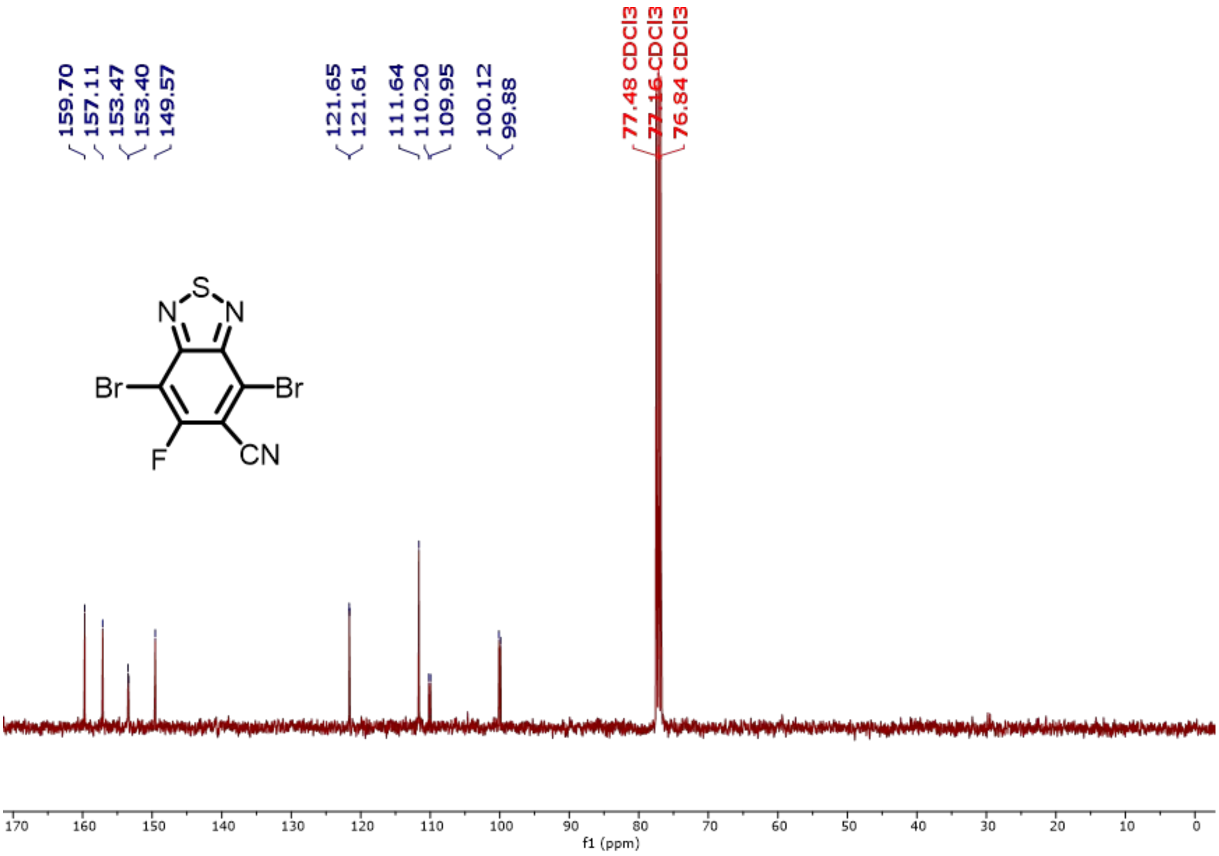

Figure S18. ^{13}C NMR spectrum of monomer FCNBT in chloroform.

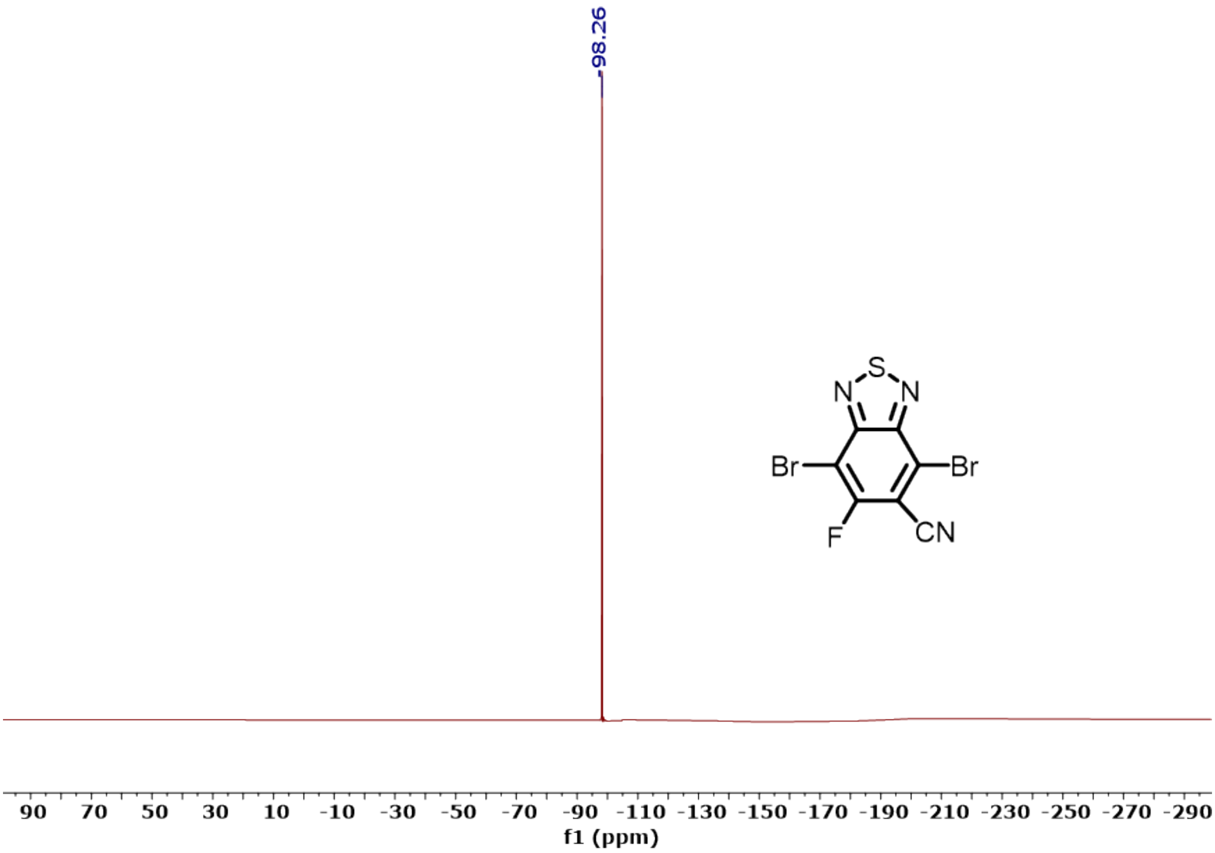

Figure S19. ^{19}F NMR spectrum of $FCNBTBr_2$.

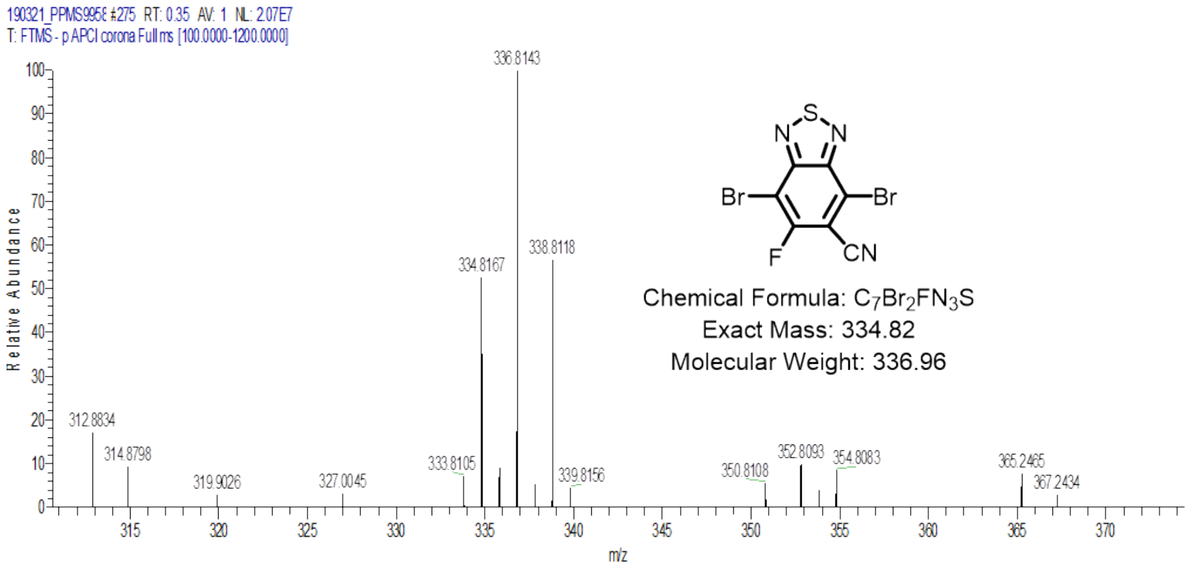

Figure S20. Mass spectrum of $FCNBTBr_2$.

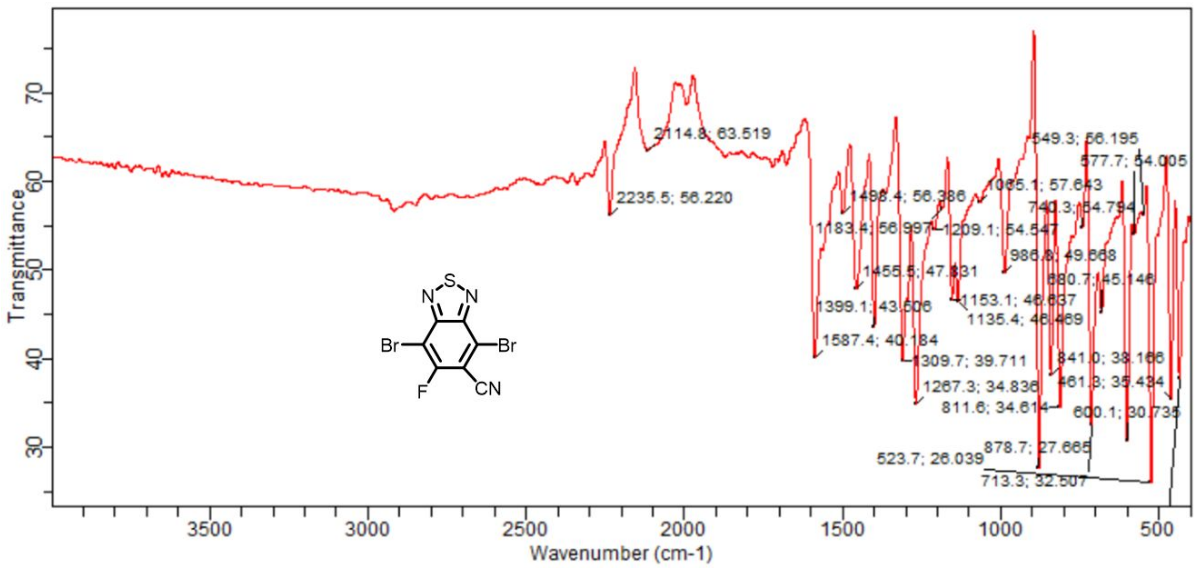

Figure S21. FTIR spectrum of $FCNBTBr_2$.

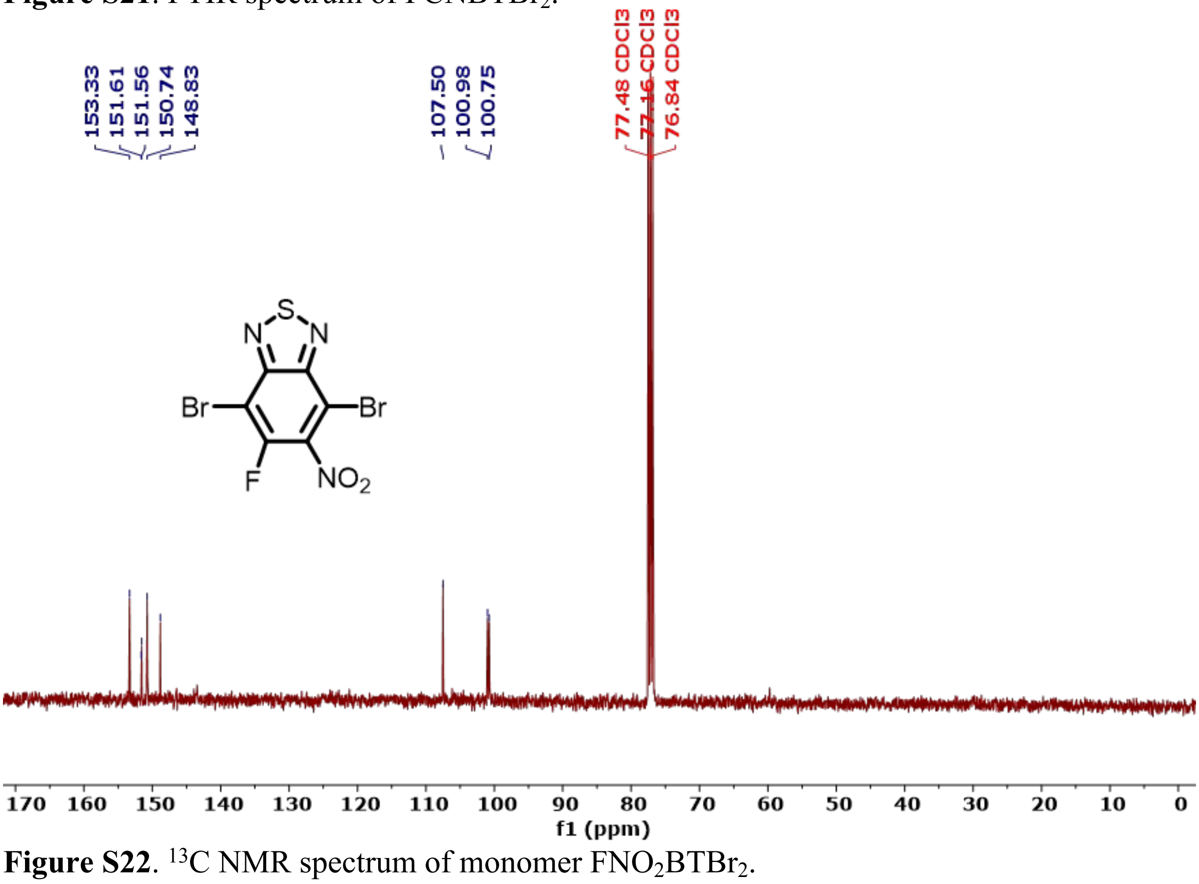

Figure S22. ^{13}C NMR spectrum of monomer FNO_2BTBr_2.

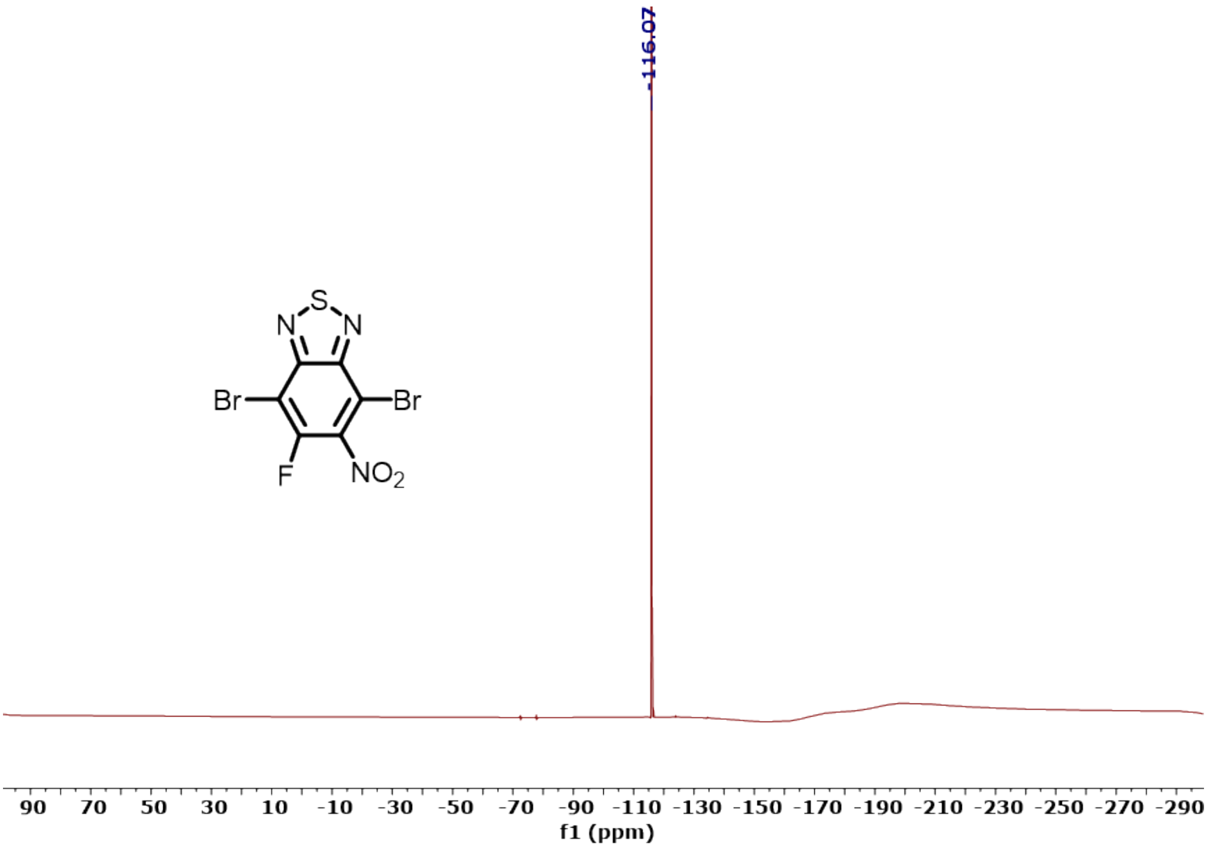

Figure S23. ^{19}F NMR spectrum of FNO_2BTBr_2.

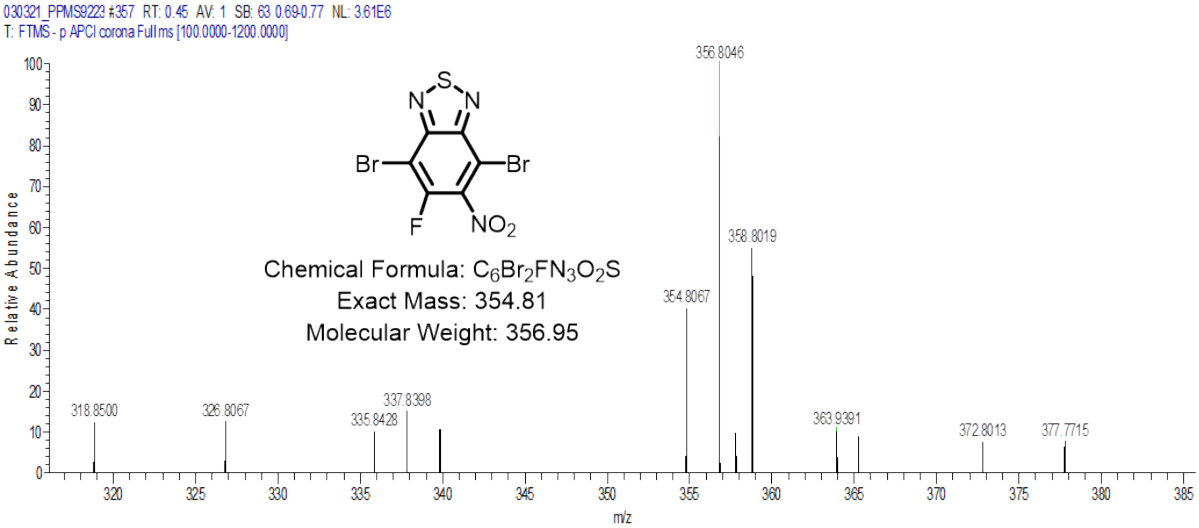

Figure S24. Mass spectrum of FNO_2BTBr_2.

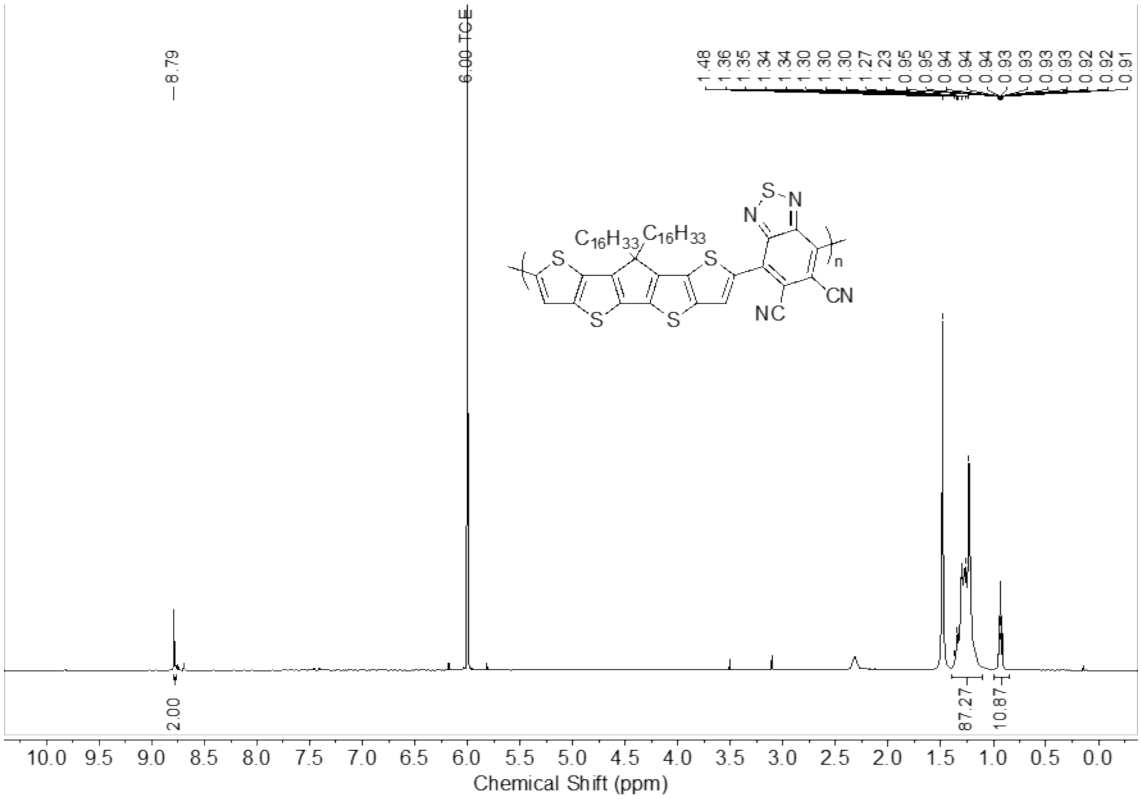

Figure S25. ^{1}H NMR spectrum of polymer **PCDTT-DCNBT** in TCE-d$_2$ at 373 K.

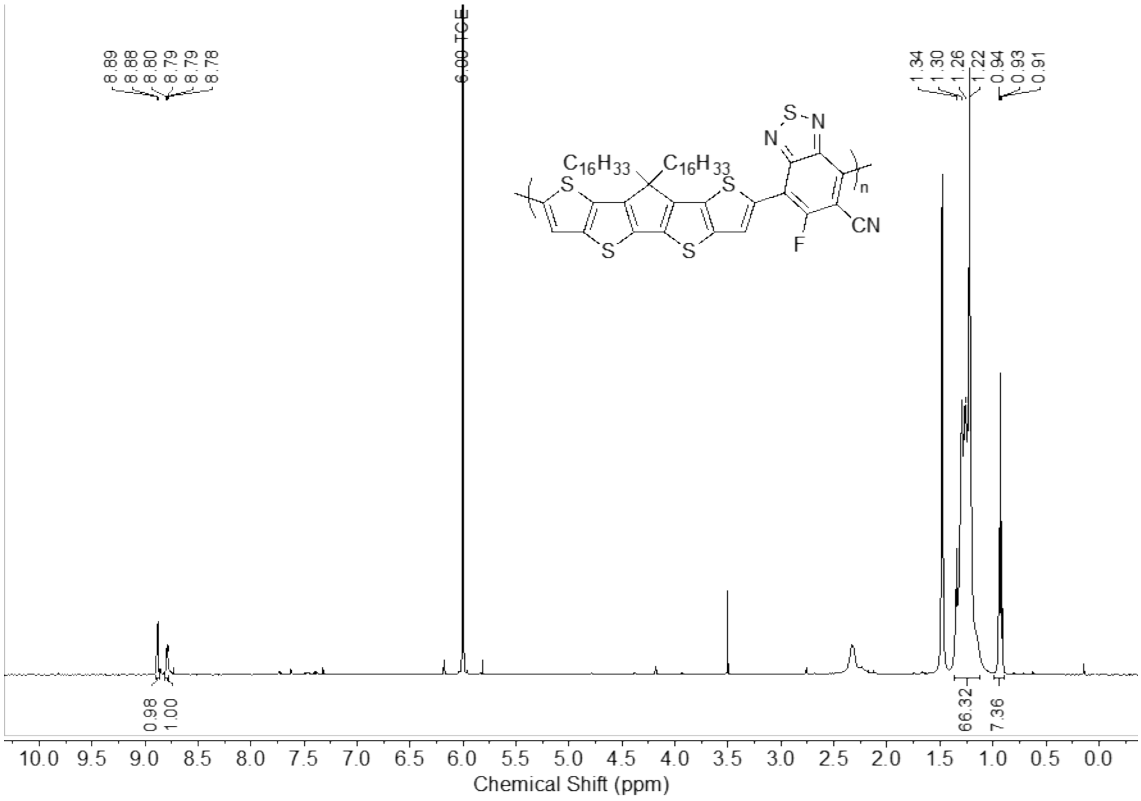

Figure S26. ^{1}H NMR spectrum of polymer **PCDTT-FCNBT** in TCE-d$_2$ at 373 K.

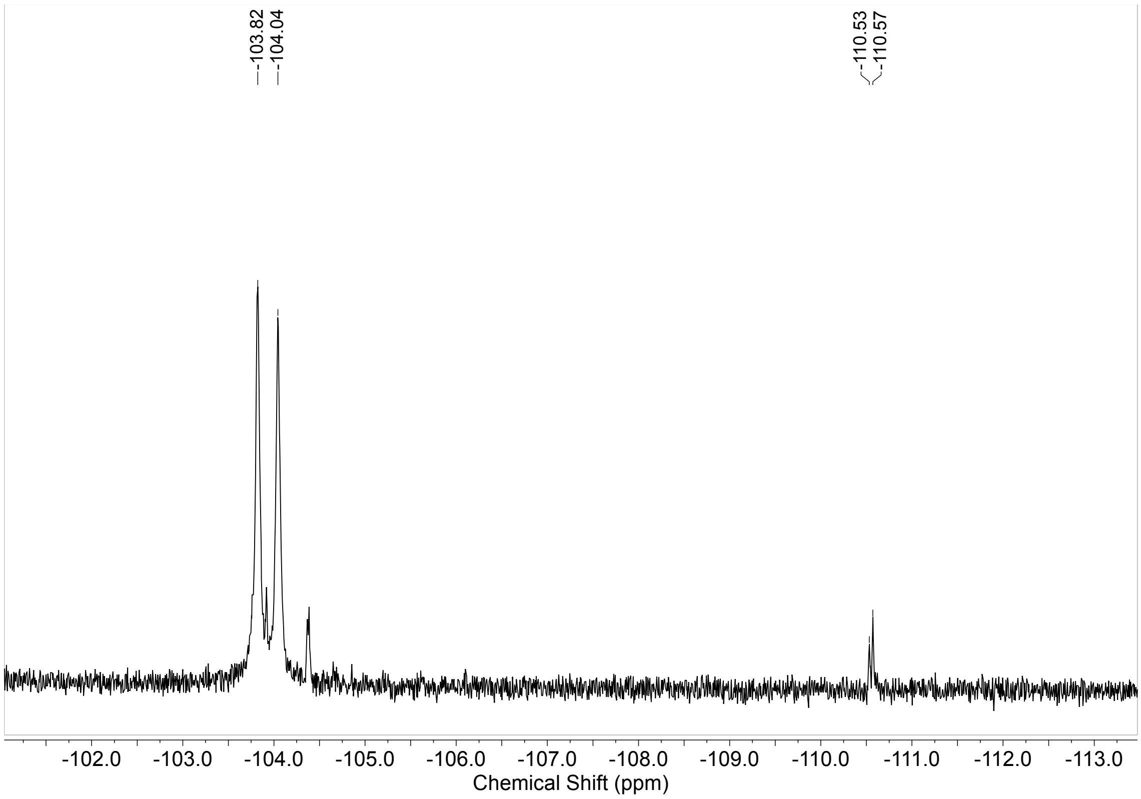

Figure S27. ^{19}F NMR spectrum of polymer **PCDTT-FCNBT** in TCE-d_2 at 373 K.

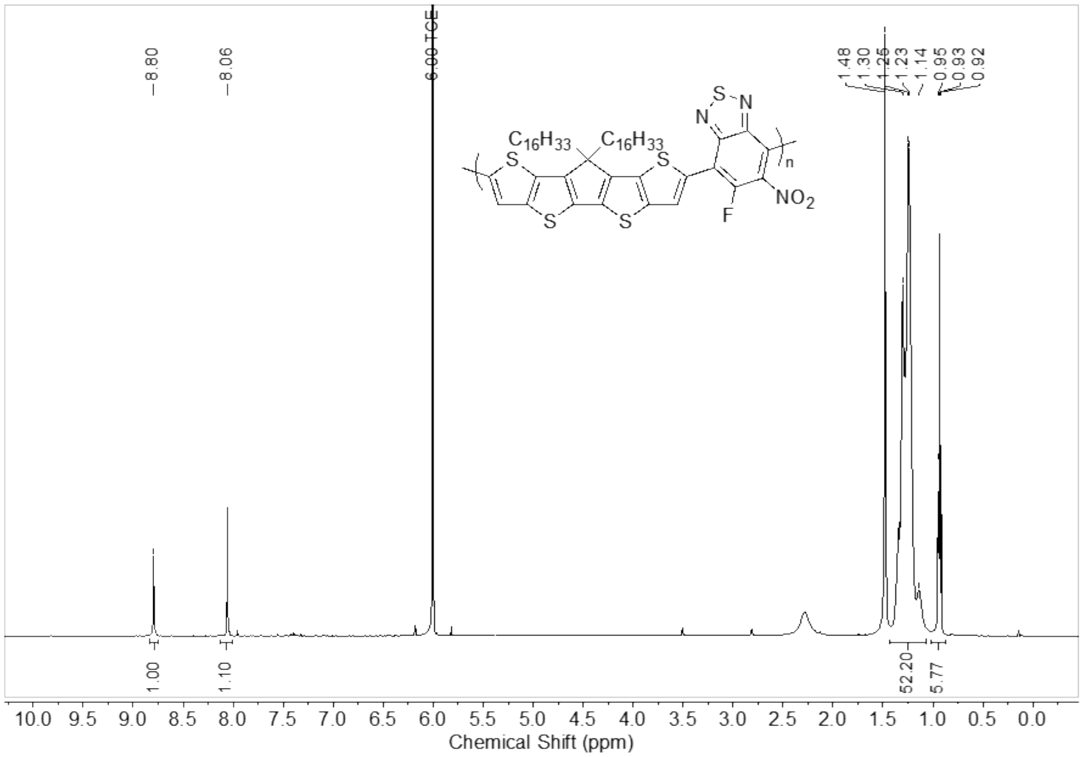

Figure S28. ^{1}H NMR spectrum of polymer **PCDTT-NO$_2$FBT** in TCE-d_2 at 373 K.

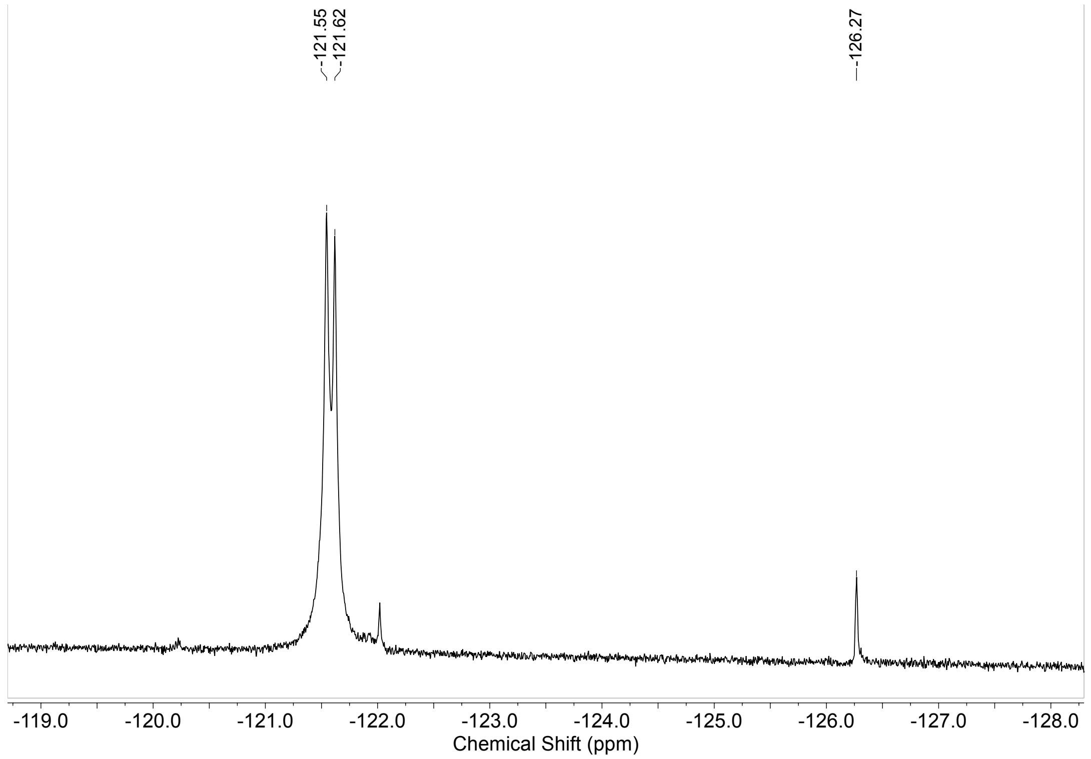

Figure S29. ^{19}F NMR spectrum of polymer **PCDTT-NO$_2$FBT** in TCE-d_2 at 373 K.

12. References

(1) Bard, A. J.; Faulkner, L. R. *Electrochemical methods : fundamentals and applications*; John Wiley, 2001.
(2) Koopmans, T. Über die Zuordnung von Wellenfunktionen und Eigenwerten zu den Einzelnen Elektronen Eines Atoms. *Physica* **1934**, *1* (1), 104-113.
(3) *Gaussian 16 Rev. C.01*; Wallingford, CT, 2016.
(4) Becke, A. D. Density-functional thermochemistry. III. The role of exact exchange. *The Journal of Chemical Physics* **1993**, *98* (7), 5648-5652.
(5) Petersson, G. A.; Al-Laham, M. A. A complete basis set model chemistry. II. Open-shell systems and the total energies of the first-row atoms. *The Journal of Chemical Physics* **1991**, *94* (9), 6081-6090.
(6) Stephens, P. J.; Devlin, F. J.; Chabalowski, C. F.; Frisch, M. J. Ab Initio Calculation of Vibrational Absorption and Circular Dichroism Spectra Using Density Functional Force Fields. *The Journal of Physical Chemistry* **1994**, *98* (45), 11623-11627.
(7) Kirby, N. M.; Mudie, S. T.; Hawley, A. M.; Cookson, D. J.; Mertens, H. D. T.; Cowieson, N.; Samardzic-Boban, V. A low-background-intensity focusing small-angle X-ray scattering undulator beamline. *Journal of Applied Crystallography* **2013**, *46* (6), 1670-1680.
(8) Ilavsky, J. Nika: software for two-dimensional data reduction. *Journal of Applied Crystallography* **2012**, *45* (2), 324-328.
(9) Wudarczyk, J.; Papamokos, G.; Margaritis, V.; Schollmeyer, D.; Hinkel, F.; Baumgarten, M.; Floudas, G.; Müllen, K. Hexasubstituted Benzenes with Ultrastrong Dipole Moments. *Angewandte Chemie International Edition* **2016**, *55* (9), 3220-3223. .
(10) Casey, A.; Green, P. J.; Shakya Tuladhar, P.; Kirkus, M.; Han, Y.; Anthopoulos, D., T; Heeney, M. Cyano substituted benzotriazole based polymers for use in organic solar cells. *J. Mater. Chem. A* **2017**, *5*, 6465-6470
